# PagMYB151 facilitates proline accumulation to enhance salt tolerance of poplar

**DOI:** 10.1186/s12864-023-09459-2

**Published:** 2023-06-22

**Authors:** Jia Hu, Shengqiang Zou, Juanjuan Huang, Xuhui Huan, Xia Jin, Lieding Zhou, Kai Zhao, Youzhi Han, Shengji Wang

**Affiliations:** 1 College of Forestry, Shanxi Agricultural University, Taigu, Shanxi 030801 China; 2 Huanghuai College, Zhumadian, Henan 463000 China

**Keywords:** PagMYB151, Poplar, Genetic transformation, Salt stress, Proline

## Abstract

Poplar is one of the main urban and rural greening and shade tree species in the northern hemisphere, but its growth and development is always restricted by salt stress. R2R3-MYB transcription factor family is commonly involved in many biological processes during plant growth and stress endurance. In this study, PagMYB151 (Potri.014G035100) one of R2R3-MYB members related to salt stress and expressed in both nucleus and cell membrane was cloned from *Populus alba × P. glandulosa* to perfect the salt tolerance mechanism. Morphological and physiological indexes regulated by PagMYB151 were detected using the *PagMYB151* overexpression (OX) and RNA interference (RNAi) transgenic poplar lines. Under salt stress conditions, compared with RNAi and the non-transgenic wild-type (WT) plants, the plant height, both aboveground and underground part fresh weight of OX was significantly increased. In addition, OX has a longer and finer root structure and a larger root surface area. The root activity of OX was also enhanced, which was significantly different from RNAi but not from WT under salt treatment. Under normal conditions, the stomatal aperture of OX was larger than WT, whereas this phenotype was not obvious after salt stress treatment. In terms of physiological indices, OX enhanced the accumulation of proline but reduced the toxicity of malondialdehyde to plants under salt stress. Combing with the transcriptome sequencing data, 6 transcription factors induced by salt stress and co-expressed with *PagMYB151* were identified that may cooperate with PagMYB151 to function in salt stress responding process. This study provides a basis for further exploring the molecular mechanism of poplar PagMYB151 transcription factor under abiotic stress.

**Supplementary Information:**

The online version contains supplementary material available at 10.1186/s12864-023-09459-2.

## Background

Poplar is one of the main urban and rural greening and shade tree species in the northern hemisphere and often planted in front and behind houses from of old. However, it’s growth and development is always restricted by salt stress due to the aggravation of soil salinization in the recent years. It is expected that this problem will be further aggravated with climate change [1, 2]. Therefore, to study the salt tolerance mechanism of poplar and improve its salt tolerance has become an urgent matter [3].

Plants have evolved adaptive mechanisms to cope with salt stress, including molecular and physiological mechanisms. At the molecular level, many genes related to salt stress response are differentially expressed after plants perceive external salt stress signals, and then the differential expression of these genes drives salt stress response at physiological and biochemical levels [4]. Transcriptional regulation is one of the important ways for plants to adapt to stress. Transcription factor (TF) regulates the transcription and expression of downstream genes by binding to potential DNA sites [5]. The main transcription factor families in plants, such as MYB, WRKY, NAC and AP2/ERF, are key regulators related to salt stress and play an important role in regulating plant response to salt stress [6]. For example, PagERF16 regulates lateral root proliferation of 84 K poplar in response to salt stress [7]. PagWRKY75 could reduce the ability of active oxygen scavenging and proline accumulation and the water holding capacity of leaves to negatively regulate the tolerance of poplar to salt and osmotic stress [8]. ERF194 overexpression poplar have higher water potential, superoxide dismutase (SOD), catalase (CAT) and peroxidase (POD) activities than wild-type under drought stress. Moreover, ERF194 up-regulates the expression of oxidoreductase and metabolism-related genes to positively regulates drought tolerance of poplar [9, 10].

As one of the largest transcription factor families in plants, MYB TFs are involved in a variety of biological processes [11]. The MYB protein consists of a conserved MYB DNA binding domain with a helix-turn-helix repeat structure of about 52 amino acids. The MYB domain is composed of 1–4 R sequences and is divided into four subfamilies (MYB-related, R2R3-MYB, 3R-MYB and 4R-MYB) according to the number of R sequences [12–14]. R2R3-MYB is the most abundant protein in the MYB family and has various regulatory functions. In chrysanthemum, CmMYB2 interacts with CmBBX24 to affect gibberellin synthesis and ultimately regulates flowering [14]. In addition, R2R3-MYB TFs are widely involved in plant physiological metabolism. MYB21 regulates flavonol biosynthesis by binding to the GARE cis-element in the promoter of FLS1 [15]. MtMYB134 could promote the synthesis of various flavonol derivatives in alfalfa hairy roots [16]. PpMYB25 and PpMYB26 could synergistically regulate the accumulation of peel wax [17]. Recent studies have shown that the allelic variation of *NsMYB1* leads to anthocyanin content variance in crops and affecting fruit color [18]. It can be seen that R2R3-MYB TFs are also a key regulator of anthocyanin accumulation and tissue coloring in some plants. In addition to participating in various physiological activities of plants, R2R3-MYB family genes are also widely involved in plant response to stress. Studies have shown that *PsnMYB108* transformants could significantly improve salt stress tolerance by increasing ROS scavenging capacity and proline accumulation [19]. MhR2R3-MYB4 plays an important role in iron deficiency stress and improves iron deficiency tolerance of apple [20]. ApMYB77 could enhanced ABA-dependent drought tolerance of *Arabidopsis* [21]. PtrMYB94 is involved in ABA-dependent drought stress regulation in poplar. The increase of endogenous ABA content will lead to changes in plant lateral root development and PtrSSR1 significantly improves plant salt tolerance by integrating lateral root growth and ABA signals [22, 23]. The MYB37 transcription factor plays an important role in the regulation of ABA, drought and seed yield of *Arabidopsis* [24].

In this study, based on the screening of poplar transcriptome data, *PagMYB151* (Potri.014G035100) which was a salt stress related gene with unknown function was identified and cloned. The function of PagMYB151 in the response to salt stress was detected using the phenotypic, physiological and molecular methods with the *PagMYB151* overexpression (OX) and RNA interference (RNAi) transgenic poplar. The results showed that PagMYB151 could increase the plant height, elongate the primary roots, facilitate the accumulation of proline and reduce the malondialdehyde (MDA) toxicity to enhance salt stress tolerance of poplar.

## Experimental design, materials and methods

### Plant materials and growth conditions

*Nicotiana benthamiana* seeds were purchased from Shaanxi Breeding Biotechnologies (http://www.biobreeding.com.cn/) and soil culture seedlings and Poplar 84 K (*Populus alba* × *P. glandulosa*) tissue culture seedlings were generated in Forest Genetics and Breeding Laboratory, College of Forestry, Shanxi Agricultural University. The growth conditions were: temperature 25 ± 2℃, 16 h/8 h light/dark cycle, relative humidity 60-70%.

### Bioinformatic analysis

The amino acid sequence of R2R3 MYB TFs was obtained from the Phytozome database (https://phytozome-next.jgi.doe.gov/). Then the amino acid sequence was searched using hmmsearch (http://www.hmmer.org/) and MYB DNA-binding (PF00249) was downloaded from the Pfam database (the threshold is E-value < 1 × 10^− 5^). The GO (Gene Ontology) function annotation and enrichment analysis of the identified family genes was performed using the Gene Ontology Resource data base (http://www.geneontology.org/).

Multiple sequence alignments were performed using Clustal X1.8 as previously described [25]. An unrooted phylogenetic tree was constructed using MEGA7 with the neighbor-joining method and 1,000 bootstrap replicates [26]. Protparam program in ExPASy was used to analyze the physicochemical properties of amino acids (https://web.expasy.org/protscale/). The online software SignalP was used to predict the signal peptide of PagMYB151 protein (https://services.healthtech.dtu.dk/service.php?SignalP-5.0). The phosphorylation sites of PagMYB151 protein were predicted using the online tool NetPhos 3.1 (https://services.healthtech.dtu.dk/service.php?NetPhos-3.1). The online tool SOPMA was used to predict the secondary structure of PagMYB151 protein (https://npsa-prabi.ibcp.fr/cgi-bin/npsa_automat.pl?page=npsa_sopma.html). SWISS-MODEL was used to predict the tertiary structure of PagMYB151 protein (https://swissmodel.expasy.org/interactive). Subcellular localization prediction was performed using Cell-Ploc2.0 (http://www.csbio.sjtu.edu.cn/bioinf/Cell-PLoc-2/) tool. The promoter sequences (2 kb upstream of the translation start site) were blasted and obtained from the poplar 84 K genomes database (https://figshare.com/articles/dataset/84K_genome_zip/12369209/5). The cis-elements in promoters were predicted and determined using the PlantCRAE (http://bioinformatics.psb.ugent.be/webtools/plantcare/html/).

### Tissue-specific expression pattern of PagMYB151 based on RNA-seq

The 20-day 84 K tissue culture seedlings with similar growth and robustness were treated with 100 mM NaCl and distilled water for 24 h, respectively. The roots, stems and leaves (materials from six plants per treatment) were collected and sequenced using IIlumina Novaseq 6000 with three technical repetitions [7]. The expression level (transcripts per million reads, TPM) of *PagMYB151* in different tissues was quantitatively analyzed using the expression quantitative software RSEM1.3.1 (http://deweylab.biostat.wisc.edu/rsem/) of Majorbio.

### RNA extraction and RT-qPCR

Total RNA from the collected poplar 84 K leaves was extracted using a RNAprep Pure Plant Kit (TIANGEN, Beijing, China) as previously described [27, 28], and complementary DNA (cDNAs) were synthesized using a Fast Quant RT Kit (TIANGEN) according to the manufacturer’s instructions. Real-time quantitative polymerase chain reaction (RT-qPCR) was performed using the SYBR Green Premix Plus (TIANGEN, Beijing, China) on a Bio-Rad Real-Time PCR System with primers MYB151DL (Supplementary Table 1). *Actin* was used as the housekeeping reference gene and the relative expression level of target genes was calculated using the 2^−∆∆Ct^ method, defined as: ∆∆Ct = (C_t−target_ − C_t−control_)_2_ −(C_t−target_ − C_t−control_)_1_ [29].

### Vector construction and poplar transformation

The full-length coding sequence of *PagMYB151* was amplified from poplar 84 K with PagMYB151 clone primers and inserted into a pBI121-GFP vector under the control of the cauliflower mosaic virus (CaMV) 35 S promoter using primers MYB151-GFP at the *Xba*I and *Sma*I sites to generate an overexpression recombinant construct (OX). The specific 300 bp sequence of *PagMYB151* CDS was inserted into the CAM-RNAi vector to obtain the *PagMYB151* RNA interference (RNAi) construct using primers MYB151-RNAi (Supplementary Table 1).

The sequenced constructs (OX, RNAi) were introduced into *EHA105* cells with the frozen-thawing method. A leaf disk transformation method was referred to generate the *PagMYB151* transgenic poplar [30]. The expression of *PagMYB151* in transgenic (OX, RNAi) and non-transgenic poplar lines (WT) was determined using PCR and RT-qPCR of leaf tissue. The primers used for vector construction, PCR, and RT-qPCR are summarized in Supplementary Table 1.

### Morphological measurements

The tissue culture seedlings of WT, OX and RNAi with uniform growth state were selected, then the apical buds were cut and inserted into 1/2 MS medium containing 0 mmol/L and 35 mmol/L NaCl, respectively. After 35 days, the aboveground fresh weight, root fresh weight, plant height, leaf number, root length and adventitious root number of each plant were measured rapidly. At least three biological replicates were set for each line.

The 3rd functional leaves were selected to compare the morphological characteristics and water content between transgenic and non-transgenic plants. Firstly, the length, width, perimeter and area of leaves were measured using intelligent leaf area measurement system (YMJ-C, Zhejiang, China), and the fresh weight (FW) of leaves was weighed. Then, the leaves were killed at 105℃ for 15 min, dried to constant weight at 80℃, and the dry weight (DW) of leaves was weighed. Finally, the water content of leaves was calculated as:$(FW-DW)/FW$ [31].

In order to detect the morphological changes of underground parts, the attached medium was washed with deionized water after the roots were taken out. The EPSON PERFECTION V700 Pro PHOTO root scanner was used to scan picture and WinRHIZO software was used to further analyze the root index.

Statistical analysis of stomatal morphology was performed using the 4th functional leaf. Lower epidermal strips were collected for measurement of stomatal apertures using an Olympus BH-2 light microscope [32, 33].

### Physiological measurements

The 3rd to 4th functional leaves of each plant were selected to measure the physiological indexes. The nitrogen blue tetrazolium (NBT) photoreduction method was used to measure SOD activity [34]. UV spectrophotometric and guaiacol methods were used to measure CAT and POD activity, respectively [35].The contents of MDA and proline were determined referencing to the previous methods [36, 37].

### Subcellular localization and transactivation assay

The 35 S: PagMYB151-GFP and 35 S: GFP (control) were transformed into *EHA105* and the cells were propagated to an OD600 of 0.6. Subsequently, the two strains were transformed into tobacco leaves respectively using a syringe and cultured in dark for 48 h. The fluorescence images were observed and recorded using the Olympus FV1000 confocal laser scanning microscope (Japan).

To determine the transactivation activity, the encoding fragment of PagMYB151 was amplified using primers MYB151-BD by PCR and the fragment was inserted into pGBKT7 vector. Then the recombinant plasmid was introduced into yeast cells [38]. The transformed yeast strains were confirmed by PCR and then cultured on the medium of SD/-Trp and SD/-Trp/-His/-Ade/X-α-Gal agar plates at 30℃ for 3 days. The transactivation activity of PagMYB151 was estimated according to the growth phenotype of transformed yeast cells.

### Gene co-expression

Based on the transcriptome sequencing data of 6 samples (stems after salt stress treatment or control, 3 biological replicates), the DEGs (log2fold-change (|log2FC|) ≥ 5 and adjusted *P*-value <0.05 using DESeq2) were selected and analyzed for construction of the co-expression gene network with *PagMYB151* using the Majorbio platform (https://report.majorbio.com/refrna/species_general/task_id/majorbio_230569). The *spearman* method was used to analyze the correlation with coefficient 0.8. The multiple test correction method was Benjamini-Hochberg and the threshold was *padj* <0.05. Subsequently, GO (Gene Ontology) [39] and KEGG (Kyoto Encyclopedia of Genes and Genomes) [40] enrichment analysis was performed on the co-expressed gene set.

### 10. Statistical analysis

Statistical analysis was performed using SPSS 22 software, and the variance of all indicators in different lines was tested by one-way analysis of variance. *t*-test was used to analyze gene expression differences between different lines or different treatments of both the RT-qPCR and RNA-seq experiments. Significant differences were determined when *P* < 0.05.

## Result

### PagMYB151 is induced by salt stress

**Fig. 1:**
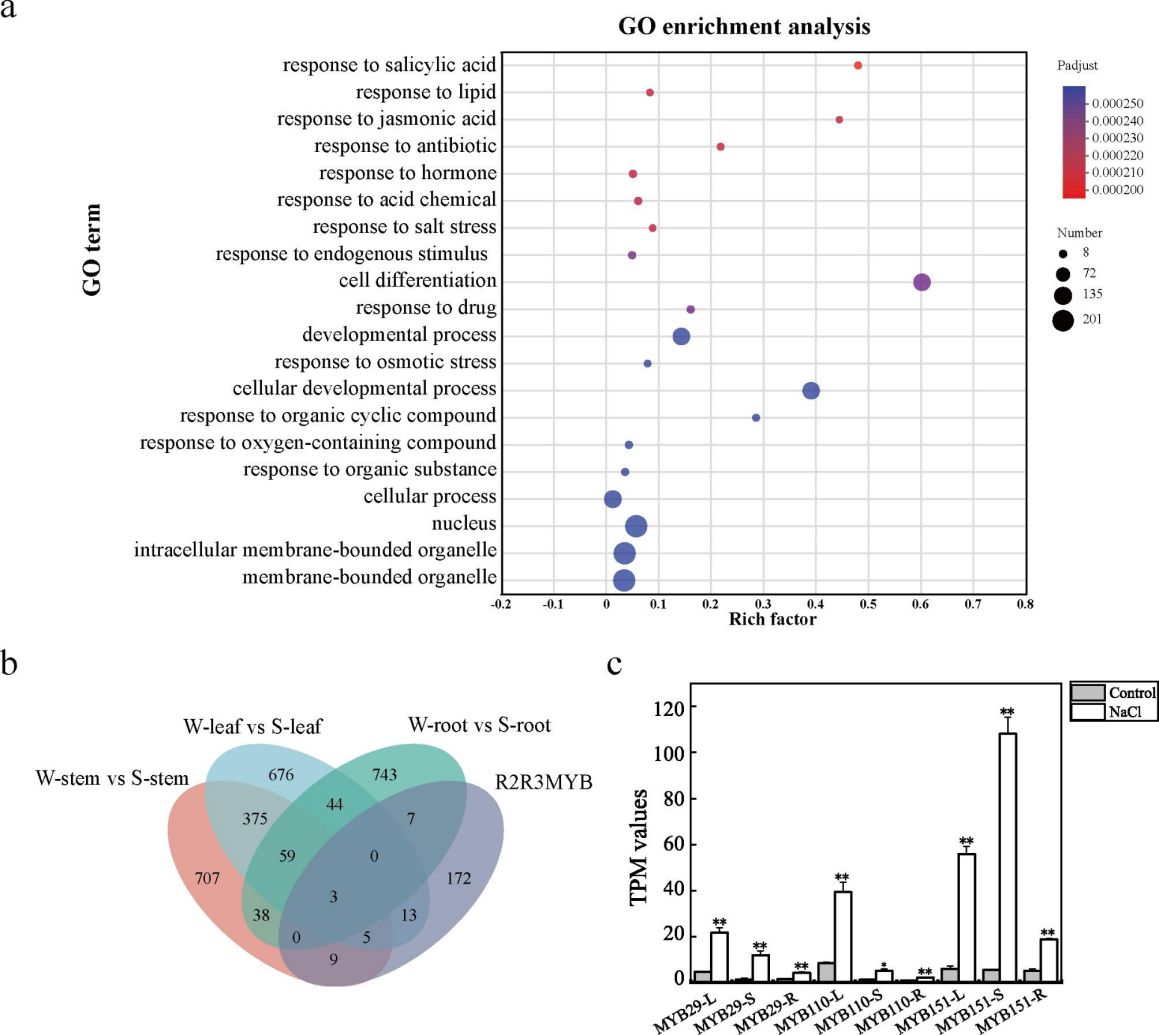
MYB151 was involved in the salt stress response process. **(a)** GO enrichment analysis and **(b)** differentially expressed genes of R2R3-MYB family in response to salt stress. **(c)** Expression level of three R2R3MYB genes in different tissues under salt stress. * represents the significant difference between samples (*P* < 0.05). ** represents the significant difference between samples (*P* < 0.01). TPM: Transcripts Per Million reads. L: leaves; S: stem; R: root; *MYB29: Potri.002G128900.1; MYB110: Potri.009G096000.1; MYB151: Potri.014G035100.1*

The amino acid sequence of poplar MYB family was searched based on *Populus trichocarpa v4.1* update database by hmmsearch software and 209 poplar R2R3-MYB family members were identified according to the number of conserved domains (Supplementary Table 2). Compared to the previous studies based on the JGI Ptri 3.0 [19], *MYB168 (Potri.015G143400), MYB190 (Potri.018G049401), MYB192 (Potri.018G058800), MYB197 (Potri.019G036160), MYB198 (Potri.019G036340) and MYB209 (Potri.T011525)* six R2R3MYB proteins were new in the *Populus trichocarpa v4.1 database*, while *Potri.003G123800, Potri.019G036300, Potri.T125000 and Potri.T144800* were omitted or replaced by a new gene ID. The results of GO annotations analysis showed that the R2R3-MYB family members were mainly related to transcriptional regulation activity and binding molecular functions, involved in organelles and cell regions cellular components, and functioned in cellular and development processes (Supplementary Fig. 1). Notably, GO enrichment analysis showed that these genes were significantly enriched in plant hormones such as salicylic acid, jasmonic acid, and salt stress responses (Fig. 1a). After salt stress treatment, 62 differentially expressed genes (DEGs) in roots, stems and leaves were identified according to RNA-seq data (Supplementary Table 3). It is worth noting that three R2R3-MYB family members including *MYB151* (Potri.014G035100) also showed significantly different expression patterns in responding to salt stress in leaf, stem and root tissues (Fig. 1b). In addition, compared with *PagMYB29 (Potri.002G128900.1)* and *PagMYB110 (Potri.009G096000.1)*, *PagMYB151* was significantly induced by salt stress in leaves, stems and roots, and the expression level in stems was about 20 times than that under the control conditions (Fig. 1c).

### Characterization of PagMYB151 transcription factor

**Fig. 2:**
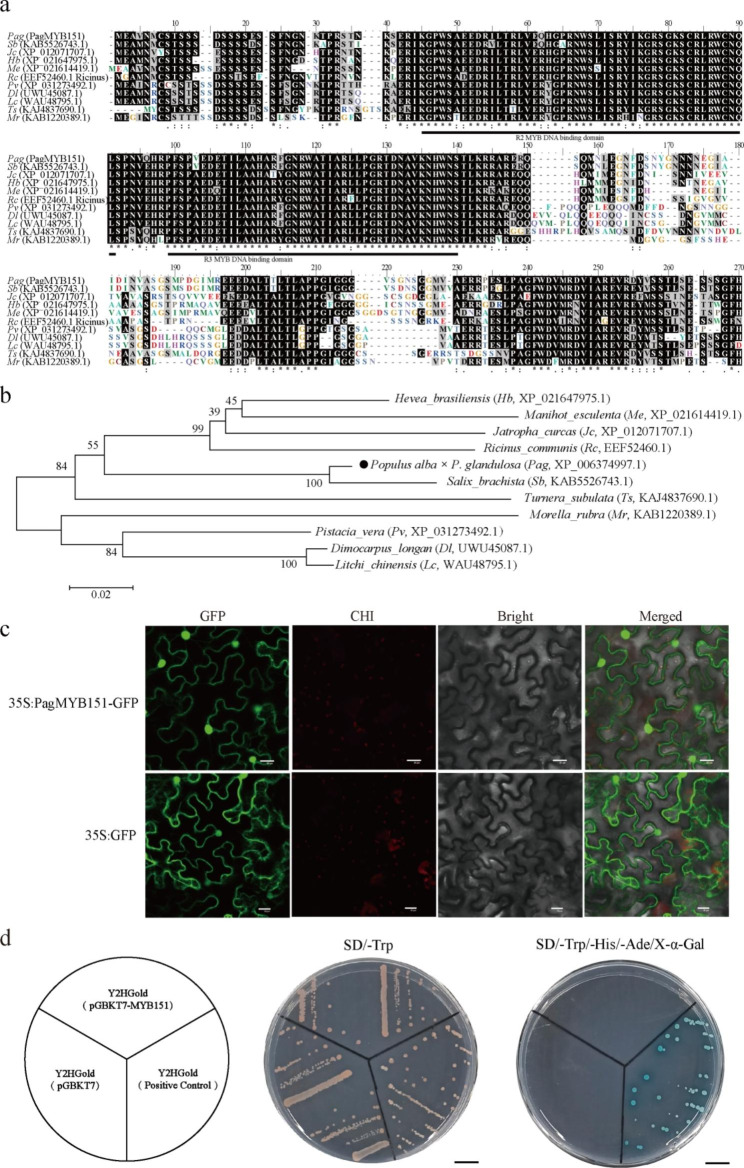
Characterization of PagMYB151 transcription factor. **(a)** Multiple sequence alignment and **(b)** phylogenetic analysis of MYB proteins of different species. **(c)** Subcellular localization of PagMYB151 protein. Bars, 20 μm. **(d)** Transcriptional activation activity analysis of PagMYB151 Bars, 1 cm

The full-length cDNA of *PagMYB151* was 744 bp, encoding 247 amino acids. The chemical formula of PagMYB151 protein was C1165H1850N368O380S10, the relative molecular mass was 27.41 kDa, the isoelectric point (pI) was 8.51, the instability coefficient was 58.44, and the total average hydrophilic index was − 0.743, which indicated that PagMYB151 was an unstable hydrophilic protein. In addition, PagMYB151 protein had 28 serine sites, 9 threonine sites, 3 tyrosine sites and no signal peptide (Supplementary Fig. 2A, B). The amino acid sequence morphology composed of PagMYB151 protein was mainly random coil and α-helix (Supplementary Fig. 2C). The amino acid sequence of PagMYB151 protein was searched by blastp in NCBI website, and ten proteins with high homology were selected for multiple sequence alignment (Fig. 2a). Results showed that these proteins had two typical DNA binding domains indicating that they were belonged to the R2R3-MYB sub-family. Phylogenetic analysis showed that PagMYB151 had the closest relationship with *Salix_brachista* (Fig. 2b). PlantCare was used for promoter cis-element analysis and the results showed that *PagMYB151* had ABRE, Box 4, CAAT-box, G-Box and other elements, that may be involved in response to low temperature, drought and abscisic acid stress (Supplementary Table 4). The PagMYB151 was mainly identified in the nucleus using Cell-PLoc2.0 online software, whereas subcellular localization analysis revealed PagMYB151 was targeted to the nucleus and cell membrane (Fig. 2c). The transactivation activity assay showed that the yeast cells containing the PGBKT7-PagMYB151 could grow normally on the SD/-Trp medium, but not on the SD/-Trp/-His/-Ade/X-α-Gal medium (Fig. 2d). This suggested that transcription factor PagMYB151 does not have self-activation ability in yeast cells and might need to be modified after translation in plants or interact with other proteins to get transcriptional activation activity and play its regulatory functions.

### PagMYB151 promotes poplar growth under salt stress

To investigate the biological function of *PagMYB151*, OX and RNAi transgenic poplar were generated under the control of a CaMV 35 S promoter (Supplementary Fig. 3A). The transgenic lines were identified by PCR and RT-qPCR (Supplementary Fig. 3B, C). A total of 7 OX lines and 8 RNAi lines were obtained. OX-2, OX-3, OX-7 with the highest expression levels and RNAi-1, RNAi-6 and RNAi-7 with the lowest expression levels were selected for further experiments.

**Fig. 3:**
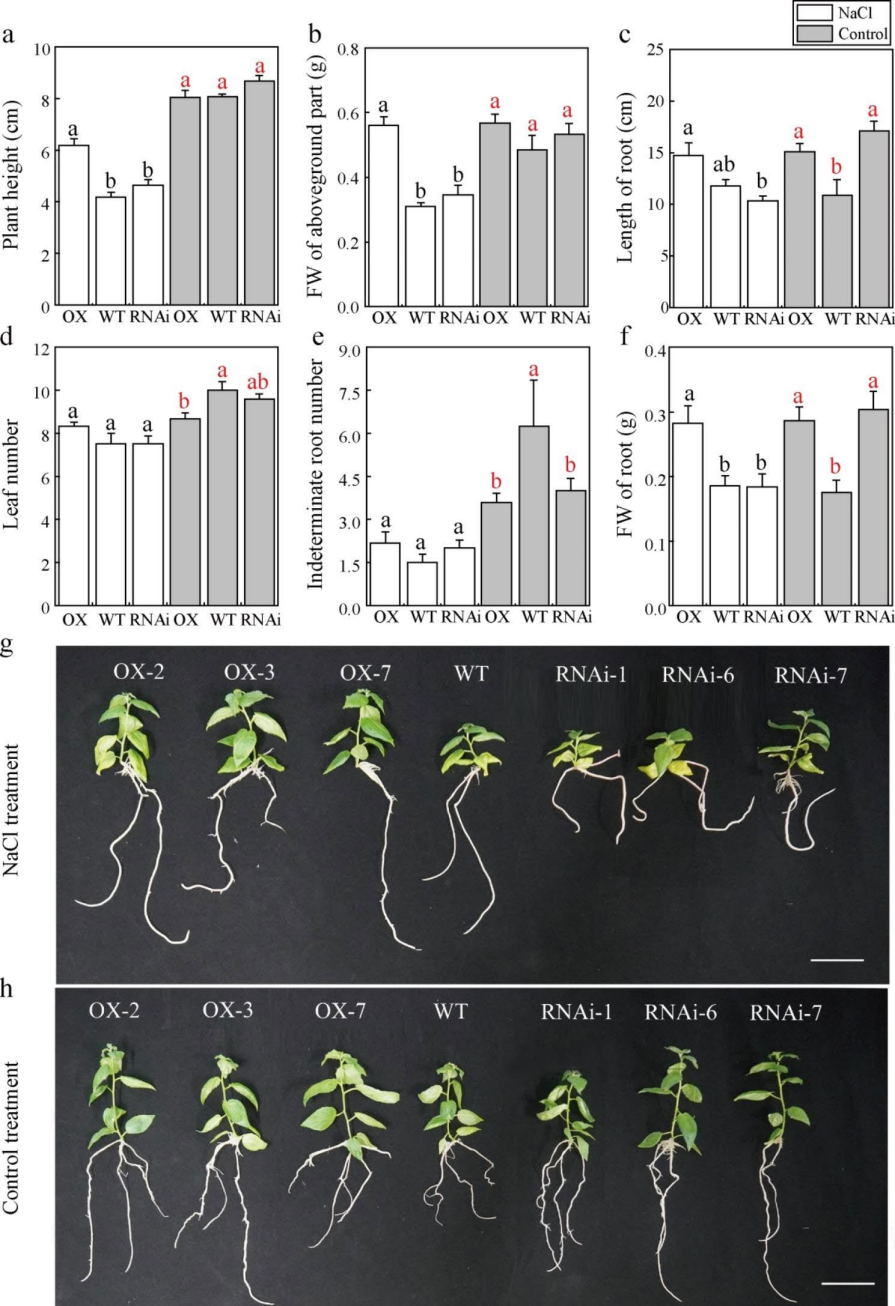
Growth indexes and phenotypes of transgenic and non-transgenic poplars under salt stress. **(a)** Plant height, **(b)** FW of aboveground part, **(c)** Length of root, **(d)** Leaf number, **(e)** Indeterminate root number, **(f)** FW of root. Morphology of WT, OX, and RNAi seedlings cultured for 35d under NaCl treatment **(g)** and control treatment **(h)**. Bars, 5 cm. Data represent the means ± SE of 12 independent biological samples of *PagMYB151* transgenic poplar (OX-2, OX-3 and OX-7, and RNAi-1, RNAi-6 and RNAi-7 served as three biological replicates, respectively.) and four biological samples of wild-type poplar, respectively. Different letters (red, control; black, NaCl treatment) indicate significant differences at the *P* < 0.05

Under normal growth conditions, there was no significant difference in plant height among OX, WT and RNAi (Fig. 3a, h). However, the plant height of OX was significantly higher than that of WT and RNAi after salt stress treatment (Fig. 3a, g). In addition, the aboveground and underground fresh weight of OX were significantly higher than WT and RNAi, which was consistent with the trend of plant height (Fig. 3b, f). Interestingly, the root length and fresh weight of RNAi were inhibited to the maximum extent under salt stress than normal conditions (Fig. 3c, f). The number of leaves and adventitious roots were also detected under salt stress though there was no significant difference among OX, WT and RNAi (Fig. 3d, e).

### PagMYB151 enlarges stomatal aperture

**Fig. 4:**
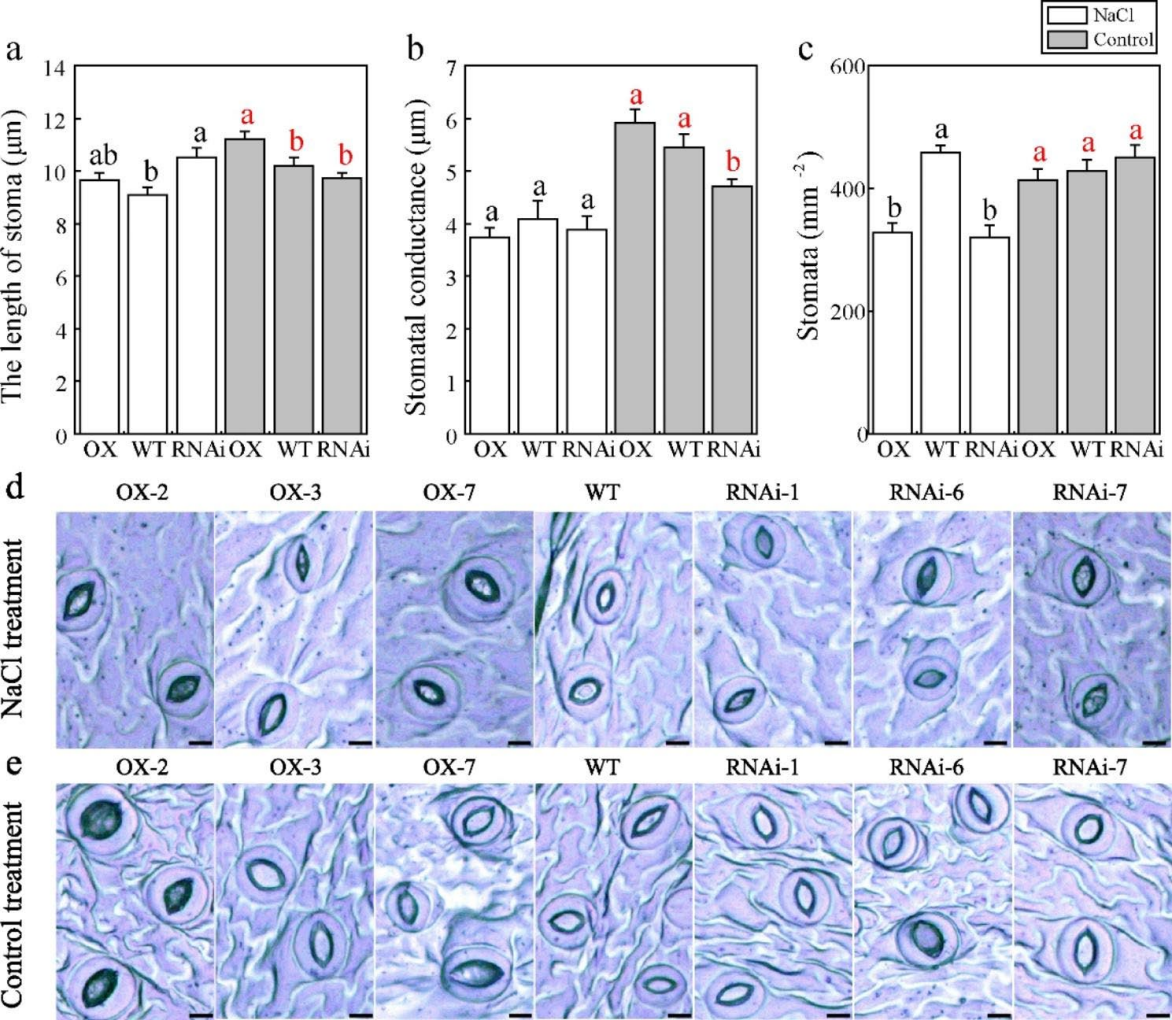
Stomatal structure of transgenic and non-transgenic poplars. **(a)** The length of stoma, **(b)** Stomatal conductance, **(c)** Density of stoma. Photos of stomatal morphology under NaCl treatment **(d)** and control treatment **(e)**. Bars, 10 μm. Data represent the means ± SE of 12 independent biological samples of *PagMYB151* transgenic poplar (OX-2, OX-3 and OX-7, and RNAi-1, RNAi-6 and RNAi-7 served as three biological replicates, respectively.) and four biological samples of wild-type poplar, respectively. Different letters (red, control; black, NaCl treatment) indicate significant differences at the *P* < 0.05

The anatomy structure photos of leaves showed that the stomatal length and stomatal conductance of OX increased significantly compared to the RNAi under normal conditions, while the stomatal density was not significantly different. In addition, the stomatal length decreased and the stomatal density increased of WT compared with OX and RNAi under salt treatment (Fig. 4). At the same time, the analysis of leaves morphological structure showed that the leaf fresh weight and dry weight of WT were significantly increased than RNAi under normal conditions (Supplementary Fig. 4a, f). However, there was no significant difference in leaf length (Supplementary Fig. 4d), leaf dry weight and leaf water content among OX, RNAi and WT under salt stress (Supplementary Fig. 4f, g), although the leaf fresh weight, leaf area, leaf perimeter and leaf width of WT were lower than those of OX and RNAi lines (Supplementary Fig. 4a, b, c, e).

### PagMYB151 elongates primary roots under salt stress

**Fig. 5:**
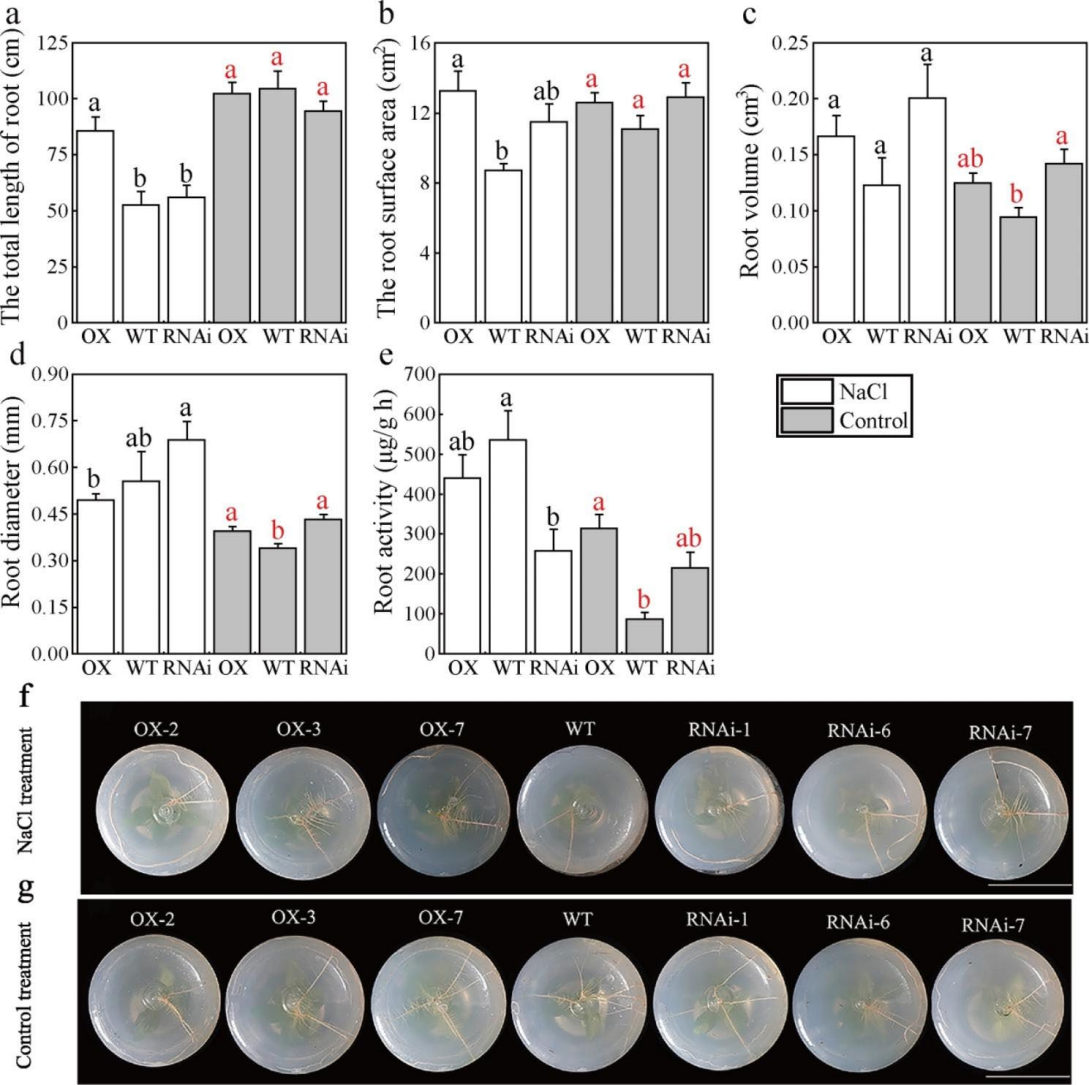
Root structure of transgenic and non-transgenic poplars. **(a)** The total length of root, **(b)** The root surface area, **(c)** Root volume, **(d)** Root diameter, **(e)** Root activity. Photos of root morphology under NaCl treatment **(f)** and control treatment **(g)**. Data represent the means ± SE of 12 independent biological samples of *PagMYB151* transgenic poplar (OX-2, OX-3 and OX-7, and RNAi-1, RNAi-6 and RNAi-7 served as three biological replicates, respectively.) and four biological samples of wild-type poplar, respectively. Different letters (red, control; black, NaCl treatment) indicate significant differences at the *P* < 0.05

WinRHIZO software was used to further analyze the root morphology, the total root length of OX increased significantly compared to WT and RNAi after salt treatment, while its root diameter decreased slightly (Fig. 5a, d). It can be seen that the significant increase in root surface area of OX may be due to the increase of its total root length (Fig. 5b). At the same time, there was no significant difference in root volume of OX, WT and RNAi after salt stress treatment (Fig. 5c). Root activity represents the growth and metabolism level of plant roots and that of OX was higher than WT and RNAi under normal growth conditions. However, the root activity of OX and WT was higher than RNAi after stress treatment (Fig. 5e). Above all, PagMYB151 showed a certain regulatory effect on the root activity of plants.

### PagMYB151 regulates stress-related physiological activity of poplar

**Fig. 6:**
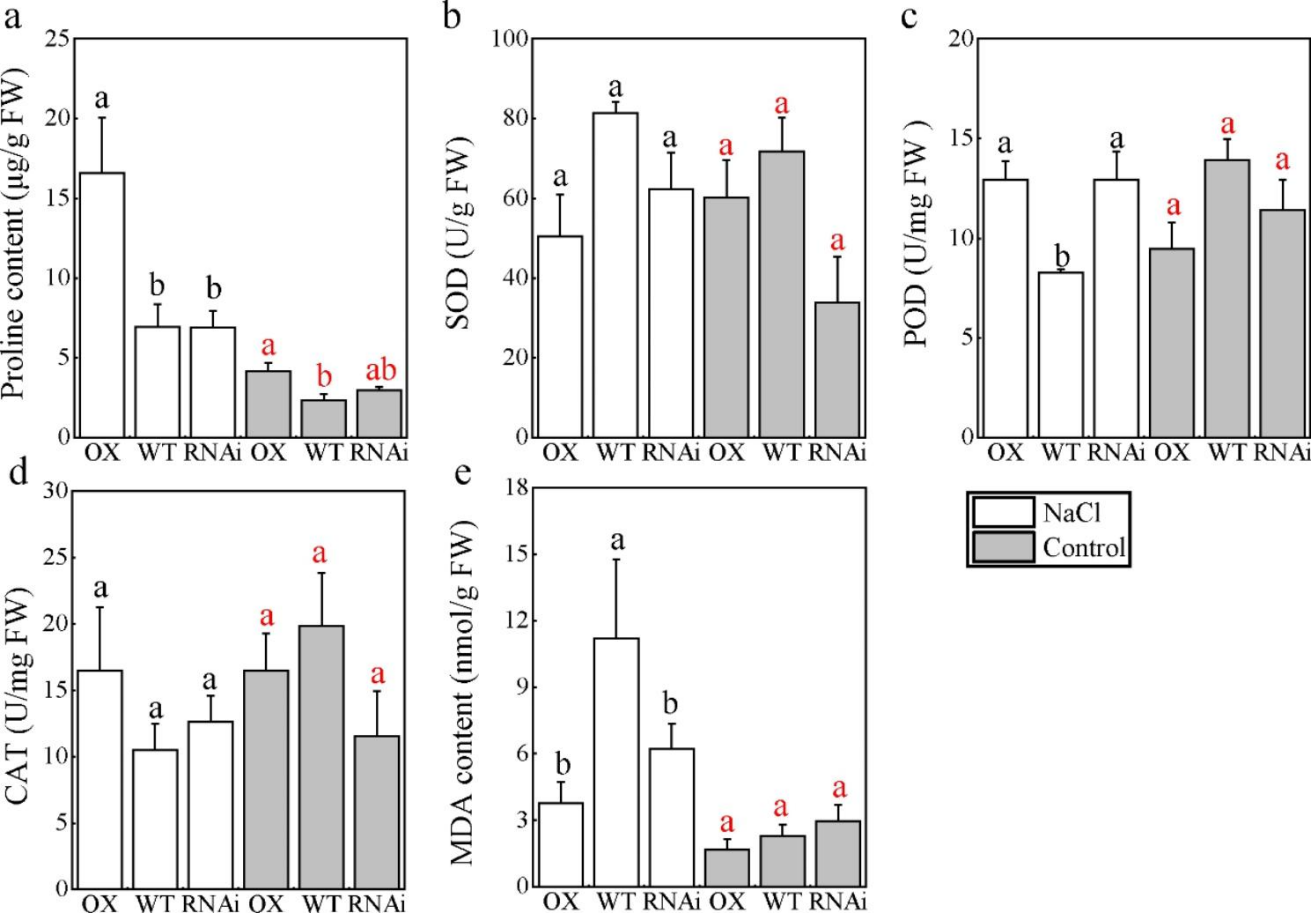
Physiological indexes of transgenic and non-transgenic poplar. **(a)** Proline content, **(b)** SOD activity, **(c)** POD activity, **(d)** CAT activity, **(e)** MDA content. Data represent the means ± SE of 12 independent biological samples of *PagMYB151* transgenic poplar (OX-2, OX-3 and OX-7, and RNAi-1, RNAi-6 and RNAi-7 served as three biological replicates, respectively.) and five biological samples of wild-type poplar, respectively. Different letters (red, control; black, NaCl treatment) indicate significant differences at the *P* < 0.05

Proline is one of the main indexes of stress induced reaction [41]. When exposed to severe environment, plants will accumulate a large amount of proline to maintain the stability of biofilm. In order to explore whether the physiological changes of plant stress resistance mediated by PagMYB151 are related to the content of proline, we compared the content of proline in different poplar lines under normal growth conditions. It can be found that the proline content in OX, WT and RNAi increased to different degrees, and the OX was significantly higher than WT and RNAi after salt treatment (Fig. 6a). These results indicated that PagMYB151 could increase the proline accumulation in plants to improve their resistance to salt stress. POD, SOD and CAT are always involved in the reactive oxygen species clearance process of plants. In this study, the SOD, POD and CAT activity of OX, RNAi and WT were not significantly different under normal conditions (Fig. 6a-d). The POD activity of WT was significantly lower than that of both OX and RNAi when treated with salt stress (Fig. 6c), suggesting that MYB151 may participate the POD biosynthesis process in more than one toles that need to be explored in the further study. MDA is one of the membrane lipid peroxidation products, which can evaluate the damage degree of cell membrane. The MDA content of OX, RNAi and WT increased after salt stress treatment. In addition, the difference between OX and WT reached a significant level and the MDA content of OX was slightly lower than that of RNAi. However, the MDA content of RNAi was significantly higher than that of WT (Fig. 6e), and the molecular mechanism behind this phenomenon needs to be further explored.

### PagMYB151 co-expresses with stress-related transcription factors

**Fig. 7:**
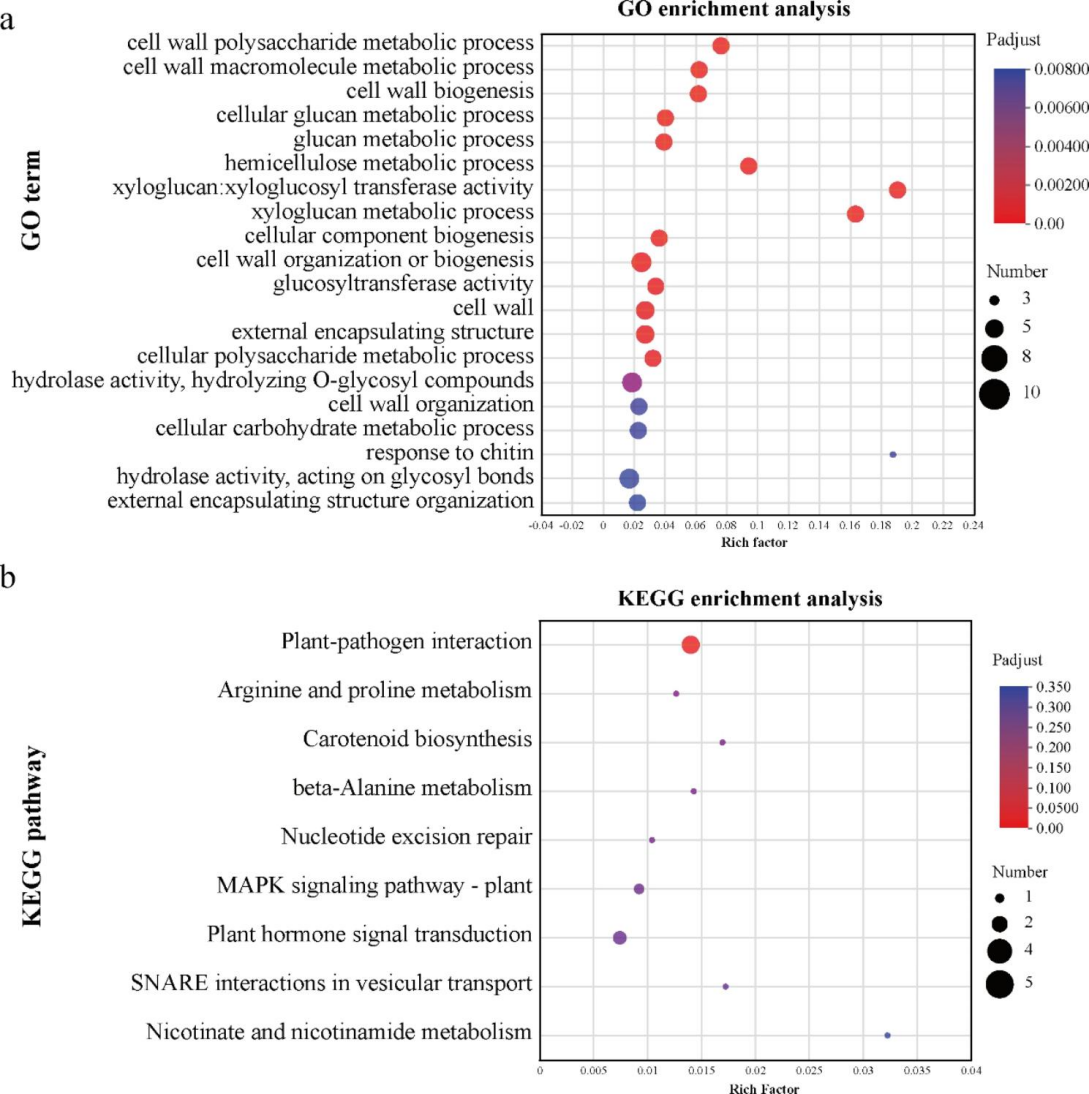
Enrichment analysis of co-expressed gene set. **(a)** GO enrichment analysis. **(b)** KEGG enrichment analysis

To obtain more information about the regulatory function of the PagMYB151, co-expression analysis was carried out to determine specific genes that may be associated with *PagMYB151*. A total of 102 poplar genes were identified to be co-expressed with *PagMYB151* (Supplementary Table 5), among which six were transcription factors belong to the ERF, GRAS and NAC family. In addition, GO enrichment analysis showed that these genes were significantly enriched in the cell wall carbohydrate metabolism process (Fig. 7a). KEGG enrichment analysis showed that these genes could be enriched in plant-pathogen metabolic pathway, arginine and proline metabolic pathway, carotenoid biosynthesis and other metabolic pathways (Fig. 7b).

**Fig. 8:**
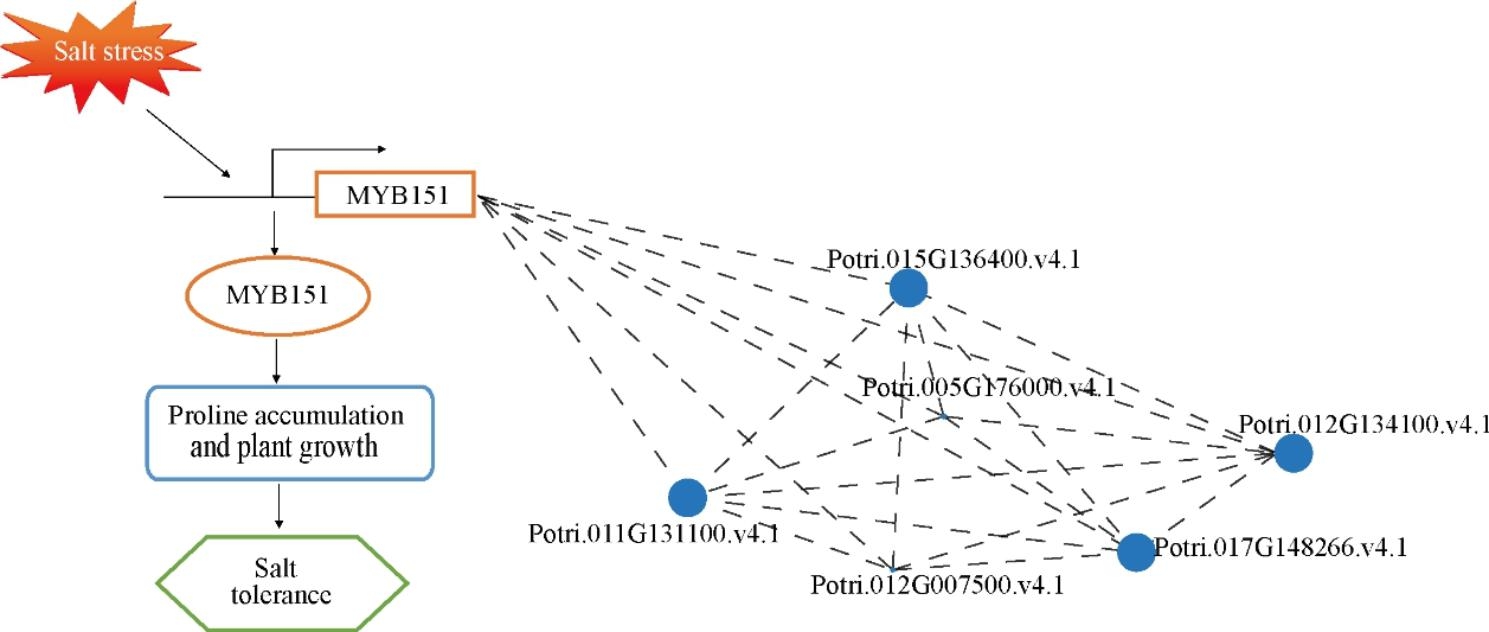
Working model diagram of PagMYB151 enhanced salt tolerance of 84 K poplar. When poplar sensed or was subjected to salt stress, *PagMYB151* and related co-expression genes were induced to facilitate the proline accumulation and promote plant growth to enhance salt tolerance

## Discussion

Salt stress is one of the major environmental stresses limiting plant growth. Plants have developed various strategies to integrate exogenous salinity stress signals with endogenous developmental cues to optimize the balance of growth and stress responses [42], such as changing the morphological, physiological and cellular processes [43].

Roots exposed to the soil directly are enable to uptake of water and dissolved nutrients efficiently [44]. Related studies have shown that transcription factors also have a certain regulatory effect on roots. *PtHDT902* could enhance primary root growth of *Arabidopsis*, but its over-expression in poplar inhibited adventitious root formation [45]. PtAIL1 is a positive regulator of poplar rooting that acts in the early development period of adventitious roots [46]. TaRNAC1 could increase root length, biomass and drought tolerance and improved grain yield under water limitation [47]. By analyzing the root phenotypic structure (Fig. 5f, g), we found that the root length and root fresh weight of OX was increased (Figs. 3f and 5a) indicating that PagMYB151 could regulate the growth structure of root to adapt to salt stress and the results of root activity also supported this conclusion. The root activity of OX and WT was higher than RNAi under salt conditions, which indicated PagMYB151 was indispensable in regulating root activity (Fig. 5e). They will sacrifice growth to survive when plants suffer from adversity. The growth index determination revealed that the growth of all poplar lines was inhibited under salt stress, but *PagMYB151* could eliminate this inhibition to a certain extent (Fig. 3b, f, g), and the increase in plant height of OX may also be attributed to the highest degree of induction of this gene in stems (Fig. 1c), indicating that PagMYB151 had a positive regulatory effect on plant growth. The difference of leaf morphology in each poplar line was not obvious (Supplementary Fig. 4), which indicated that the increase of underground biomass mainly contributed to the growth of poplar plant height. Owing to their ability to control gas exchange, stomata are essential regulators of photosynthesis and transpiration [48]. The regulation of stomatal morphology plays a key role in plant adaptation to changes in environmental conditions [49]. The larger stoma of OX was in favour of gas exchange and material accumulation under normal conditions (Fig. 4a, b). However, the stomatal density of OX and RNAi was lower than that of WT under salt stress conditions, indicating that *PagMYB151* did not regulate stomata to respond to salt stress. Above all, PagMYB151 may regulate plant height mainly by promoting root growth.

Plants adopt various endogenous strategies to resist oxidative stress caused by adversity, including oxidative defense system and osmotic accumulation [50]. As a potential penetrant, proline can scavenge free radicals produced by plants due to stress. At the same time, proline can also be used as a non-enzymatic antioxidant to participate in the oxidative defense system to neutralize ROS. In this study, we found that a large amount of proline was accumulated in OX under salt stress (Fig. 6a). The accumulation of proline is often related to the increased expression of its biosynthetic genes. For example, *TaERF87* activated the expression of the proline biosynthesis genes *TaP5CS1* and *TaP5CR1* via direct binding to GCC-box elements to enhance drought tolerance in wheat [51]. Related studies have shown that proline is actively transported to the roots to maintain root elongation under stress conditions. For example, proline accumulation caused by overexpression of *P5CSF129A* could make plants exhibit root elongation and higher root biomass both under salt and drought stress conditions [52]. In addition, the accumulation of proline also helps to improve the activity of antioxidant enzymes, making the plant’s oxidative defense system more powerful. However, the antioxidant enzyme activity of PagMYB151 overexpression lines did not show a significant increase (Fig. 6b, c, d), which may be related to the salt concentration in the rooting medium. Furthermore, previous studies have shown that proline itself could completely neutralize salt-induced oxidative stress under low salt stress [53]. Lipid peroxidation is an obvious indicator of plant injury, and the MDA content of OX is lower than that of WT and RNAi under both treatments (Fig. 6e). Therefore, PagMYB151 responds to salt stress mainly by increasing proline content in plants.

In view of the fact that OX could accumulate proline and promote the growth of poplar under salt stress, gene co-expression analysis was performed to link genes with unknown functions and biological processes. GO enrichment analysis showed that these genes were significantly enriched in the cell wall carbohydrate metabolism process. At the same time, KEGG enrichment analysis showed that these genes could be enriched in plant-pathogen metabolic pathway, arginine and proline metabolic pathway, carotenoid biosynthesis and other metabolic pathways. Proline is not only a compatible solute involved in stress resistance, but also constitutes a free amino acid with N storage function [54]. Arginine and proline can be used as nitrogen sources to supply plant growth. Previous studies have shown that proline is involved in the synthesis of cell wall proteins [55], which make the plants maintain its original morphology under osmotic stress. As an important component of cell wall protein, proline plays a key role in cell wall signal transduction cascade, plant development and stress tolerance. By searching for TFs in the co-expressed gene set, six TFs involved in a variety of biological processes were found. Among them, overexpression of *CBF4* in transgenic *Arabidopsis* results in the activation of downstream genes involved in cold acclimation and drought adaptation through binding to the C-repeat/dehydration-responsive element [56]. *DREB26* plays a role in salt and osmotic stress tolerance of *Arabidopsis* [57]. Turnip crinkle virus coat protein inhibits the basal immune response to virus invasion in *Arabidopsis* by binding to the TIP transcription factor [58]. GAF1 can participate in gibberellin signaling pathway and plays an important role in plant growth and development [59]. On these grounds, PagMYB151 was likely to be involved in these process to enhance the adaptability of plants in stress environment, which will be a direction for our future exploration.

## Conclusion

In conclusion, salt stress induced the expression of *PagMYB151* (Fig. 8). Overexpressed *PagMYB151* promoted poplar growth by changing root structure, facilitated the accumulation of proline and decreased the content of MDA under salt stress. By regulating the above morphological, physiological and molecular processes, PagMYB151 functioned with the co-expression transcription factor to enhanced salt tolerance of 84 K poplar These findings will provide new ideas for the physiological functions of the PagMYB151 transcription factor in plant growth and salt stress tolerance.

## Electronic supplementary material

Below is the link to the electronic supplementary material.

Supplementary Material 1

Supplementary Material 2

Supplementary Material 3

Supplementary Material 4

Supplementary Material 5

Supplementary Material 6

## Data Availability

All data generated or analysed during this study are included in this published article and its supplementary information files. The raw sequencing data used during this study has been deposited in NCBI SRA with the accession number PRJNA716488. The XM_ 006374935.3 is used as the sequence ID of MYB151 (*Potri.014G035100)* in Genbank. Competing interests. The authors declare no competing interests.

## Abbreviations

TF: Transcription factor
OX: Overexpression transgenic poplar lines
RNAi: RNA interference poplar lines
WT: Non-transgenic wild-type poplar
RT-qPCR: Real-time-quantitative polymerase chain reaction
SOD: Superoxide dismutase
CAT: Catalase
POD: Peroxidase
MDA: Malondialdehyde
DEGs: Differentially expressed genes
GO: Gene Ontology
KEGG: Kyoto Encyclopedia of Genes and Genomes
