# Supplementary figures and images for: PagMYB151 facilitates proline accumulation to enhance salt tolerance of poplar
Source: BMC Genomics. 2023 Jun 22;24:345. doi: 10.1186/s12864-023-09459-2 (PMC10286439; doi:10.1186/s12864-023-09459-2)

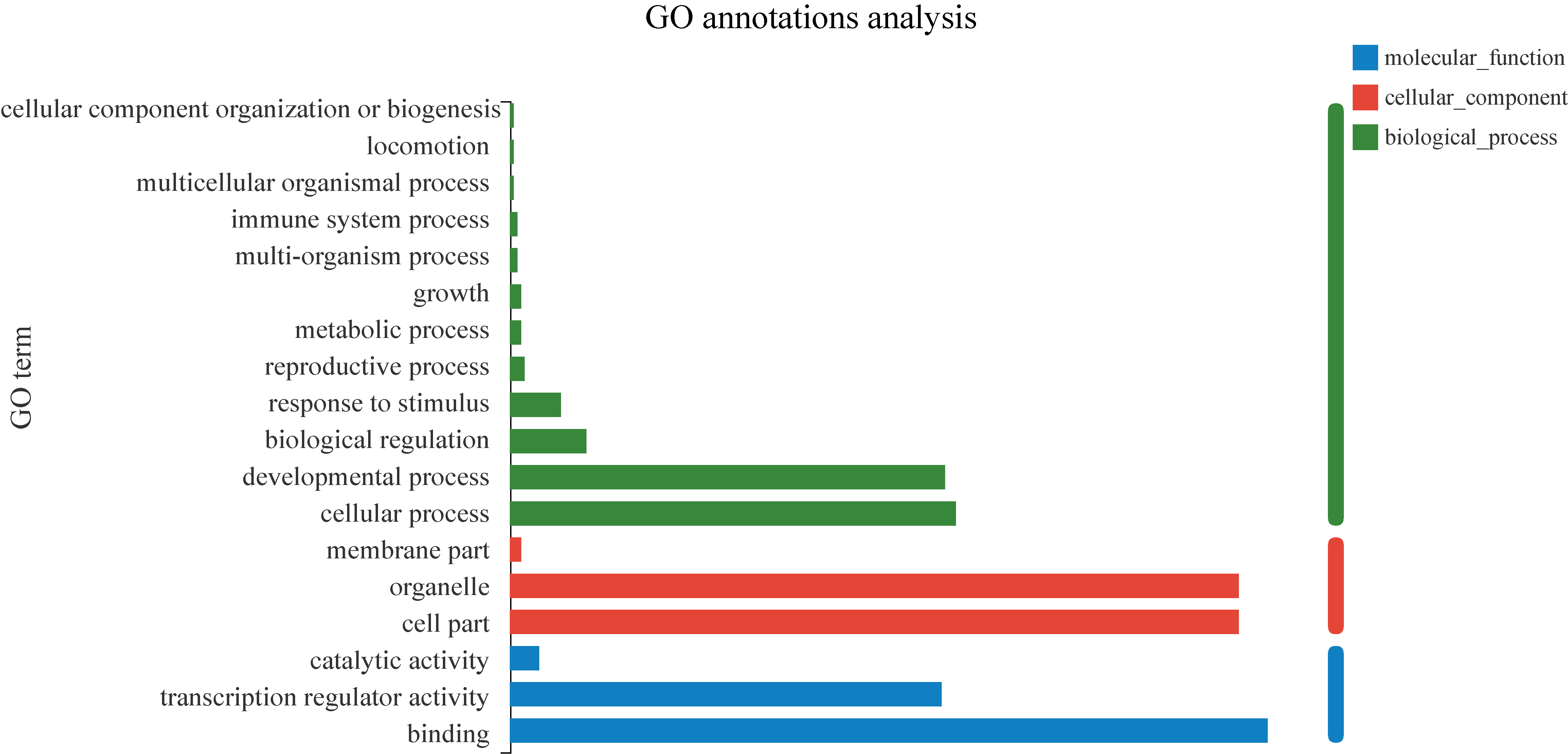

Supplement: Supplementary file 1 — Supplementary Material 1 [file 12864_2023_9459_MOESM1_ESM.tif]

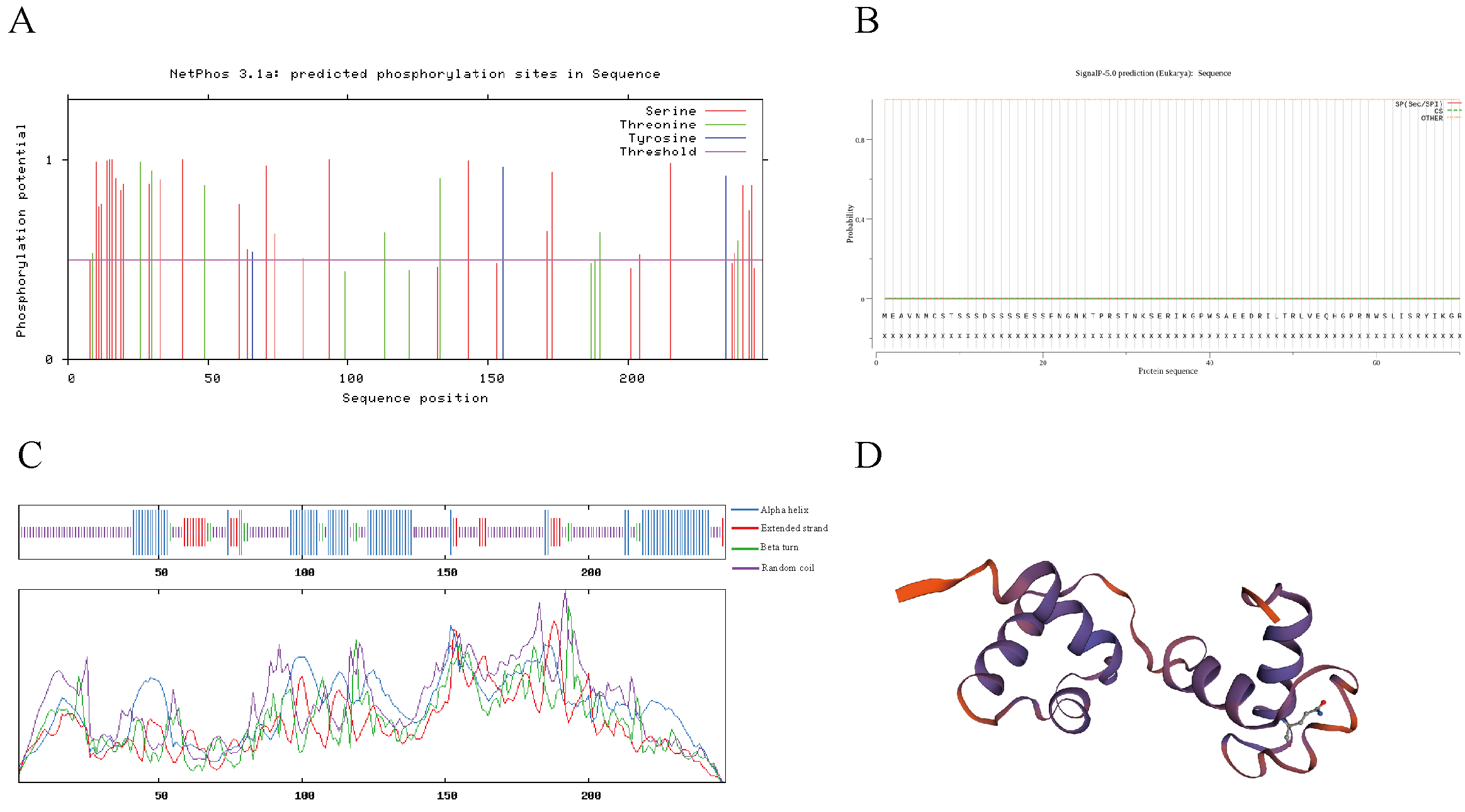

Supplement: Supplementary file 2 — Supplementary Material 2 [file 12864_2023_9459_MOESM2_ESM.tif]

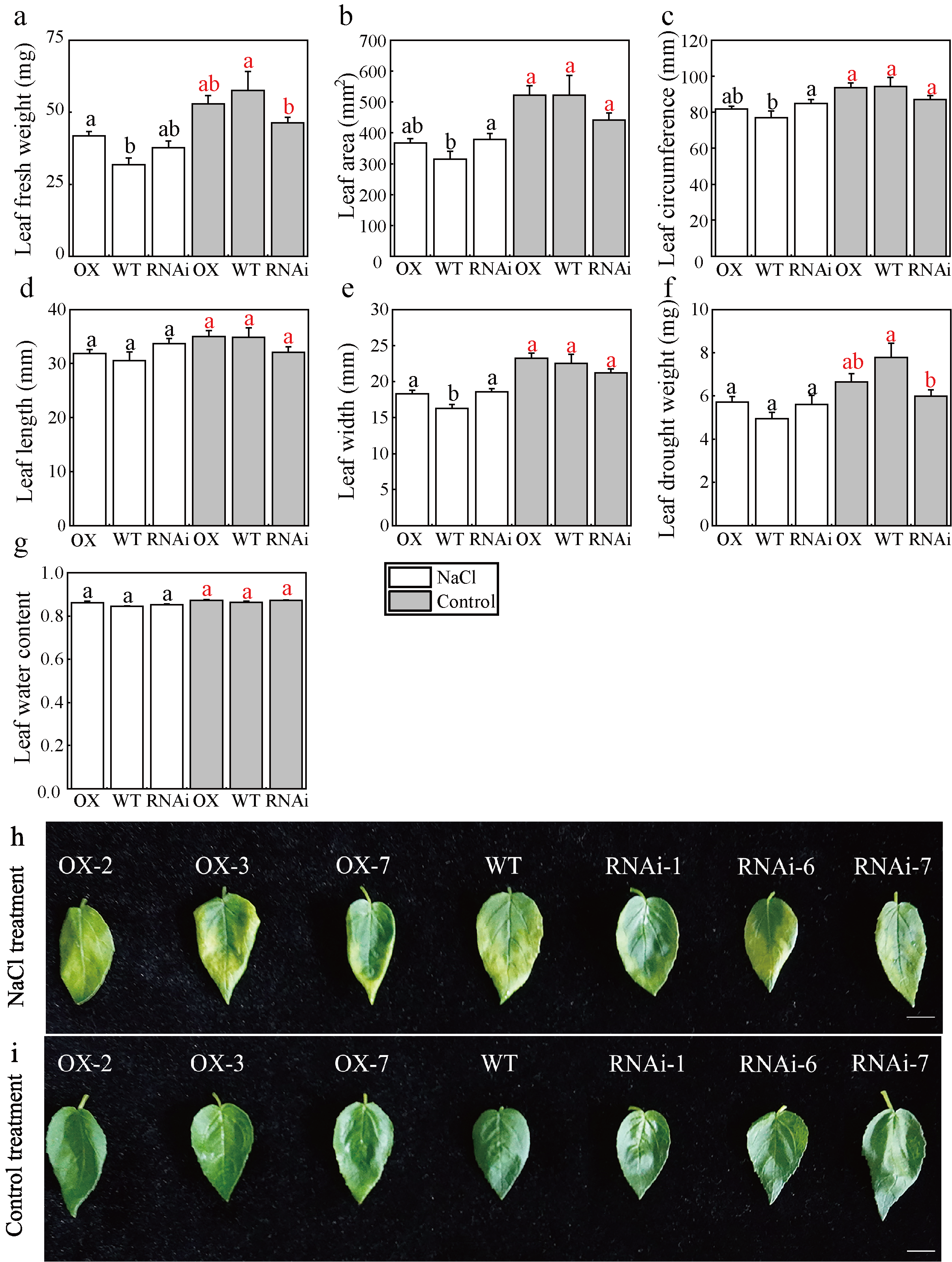

Supplement: Supplementary file 3 — Supplementary Material 3 [file 12864_2023_9459_MOESM3_ESM.tif]

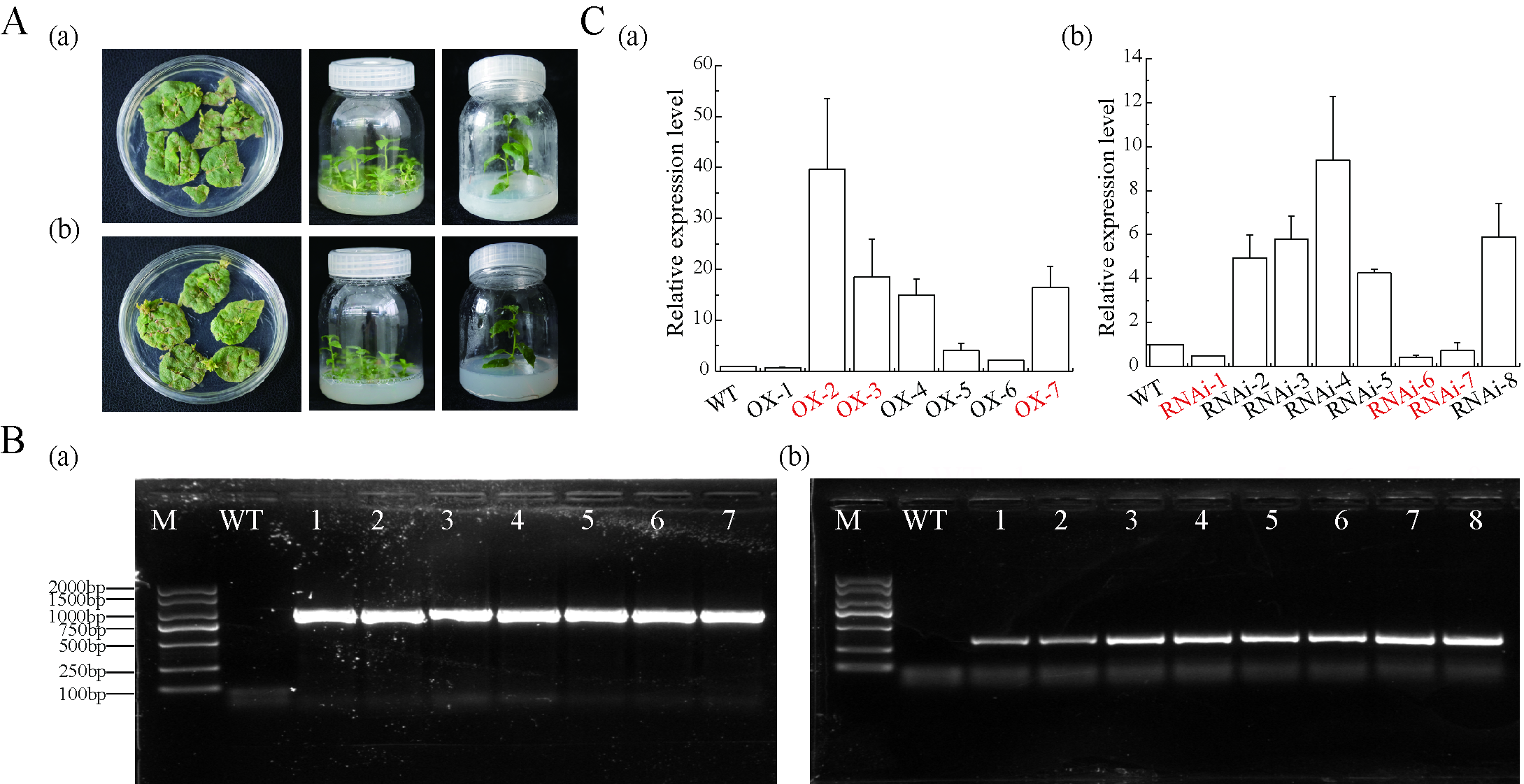

Supplement: Supplementary file 4 — Supplementary Material 4 [file 12864_2023_9459_MOESM4_ESM.tif]
