# Supplementary material for: PagMYB151 facilitates proline accumulation to enhance salt tolerance of poplar
Source: BMC Genomics. 2023 Jun 22;24:345. doi: 10.1186/s12864-023-09459-2 (PMC10286439; doi:10.1186/s12864-023-09459-2)
Supplement: Supplementary file 5 — Supplementary Material 5 [file 12864_2023_9459_MOESM5_ESM.docx]

Supplementary Table 1 Design of primers

| Name | Sequence(5＇-3＇) | Length/bp |
| --- | --- | --- |
| MYB151-F | TTATGGAAGCAGTGAACATG | 20 |
| MYB151-R | GTCCCAACTAATGAAATCCTGAGC | 24 |
| MYB151-GFP-F | CTAGTCTAGAATGGAAGCAGTGAACATGTGC | 31 |
| MYB151-GFP-R | CTAGCCCGGGTAATGAAATCCTGAGCTACTCTC | 33 |
| PBI121-F | CCATCGTTGAAGATGCCTCTGC | 22 |
| PBI121-R | CTCTTCGCTATTACGCCAGCTG | 22 |
| MYB151-BD-F | CTAGGAATTCATGGAAGCAGTGAACATGTGC | 31 |
| MYB151-BD-R | CTAGGTCGACCTAATGAAATCCTGAGCTACTCTC | 34 |
| pGBKT7-F | TCATCGGAAGAGAGTAGT | 18 |
| pGBKT7-R | GAGTCACTTTAAAATTTGTATA | 22 |
| MYB151 RNAi F-F | ATTTGGAGAGGACACGCTCGAGCATGGACCCAGAAACTGG | 40 |
| MYB151 RNAi F-R | ACCAAGCTGGGGTACCGAATTCGTTACTATCAAAATTACCTT  CCAAATTC | 50 |
| MYB151 RNAi R-F | TGGGTTCGAAATCGATAAGCTTGTTACTATCAAAATTACC  TTCCAAATTC | 50 |
| MYB151 RNAi R-R | CTCATTAAAGCAGGACTCTAGACATGGACCCAGAAACTGG | 40 |
| Actin-F | ACCCTCCAATCCAGACACTG | 20 |
| Actin-R | TTGCTGACCGTATGAGCAAG | 20 |
| MYB151DL-F | GTAGCTTCAGGATCTATGCCGGATG | 25 |
| MYB151DL-R | GCAATAACATCCCTCATCACATC | 23 |

Supplementary Table 2 List of poplar R2R3-MYB family members

| Gene ID | Gene ID | Gene ID | Gene ID |
| --- | --- | --- | --- |
| Potri.001G005100.1  Potri.001G035800.1  Potri.001G036000.1  Potri.001G075400.1  Potri.001G086700.1  Potri.001G099800.1  Potri.001G118800.1  Potri.001G139900.1  Potri.001G169600.1  Potri.001G197000.1  Potri.001G219100.1  Potri.001G224500.2  Potri.001G235500.1  Potri.001G248800.2  Potri.001G250000.1  Potri.001G258700.1  Potri.001G267300.1  Potri.001G300200.1  Potri.001G336700.1  Potri.001G346600.4  Potri.001G347200.1  Potri.001G408700.1  Potri.001G470500.1  Potri.002G038500.1  Potri.002G073500.1  Potri.002G096800.1  Potri.002G113700.2  Potri.002G122600.1  Potri.002G128900.1  Potri.002G140900.1  Potri.002G157600.1  Potri.002G173900.1  Potri.002G185900.1  Potri.002G191800.2  Potri.002G198100.1  Potri.002G228700.1  Potri.003G064600.1  Potri.003G079100.1  Potri.003G094200.1  Potri.003G114100.1  Potri.003G132000.2  Potri.003G144200.1  Potri.003G144300.1  Potri.003G155700.1  Potri.003G168900.1  Potri.003G189700.7  Potri.003G219900.1  Potri.004G026600.1  Potri.004G033100.1  Potri.004G086300.1  Potri.004G088100.1  Potri.004G102600.1  Potri.004G115600.1 | Potri.004G118000.1  Potri.004G126700.1  Potri.004G138000.1  Potri.004G174400.1  Potri.004G215100.3  Potri.005G001600.1  Potri.005G063200.1  Potri.005G074500.1  Potri.005G087700.2  Potri.005G096600.1  Potri.005G112000.2  Potri.005G118500.1  Potri.005G142600.1  Potri.005G164900.1  Potri.005G186400.1  Potri.005G224100.1  Potri.006G066400.1  Potri.006G085900.1  Potri.006G097300.1  Potri.006G122100.1  Potri.006G123400.1  Potri.006G170800.1  Potri.006G221200.1  Potri.006G221500.1  Potri.006G221800.1  Potri.006G234200.1  Potri.006G275900.2  Potri.007G007900.1  Potri.007G048900.5  Potri.007G064600.2  Potri.007G067600.1  Potri.007G076200.10  Potri.007G093900.1  Potri.007G106100.3  Potri.007G134500.1  Potri.008G062700.1  Potri.008G064200.1  Potri.008G070900.1  Potri.008G081600.2  Potri.008G088000.1  Potri.008G089200.1  Potri.008G089700.1  Potri.008G101400.1  Potri.008G122100.1  Potri.008G128500.1  Potri.008G148400.1  Potri.008G166700.1  Potri.008G173400.1  Potri.008G180800.1  Potri.009G007100.1  Potri.009G018700.1  Potri.009G027300.1  Potri.009G042600.1 | Potri.009G044100.1  Potri.009G053900.1  Potri.009G061500.1  Potri.009G096000.1  Potri.009G134000.1  Potri.010G004300.1  Potri.010G064000.1  Potri.010G093000.1  Potri.010G114000.1  Potri.010G123000.1  Potri.010G141000.1  Potri.010G149900.1  Potri.010G165700.1  Potri.010G167500.2  Potri.010G174500.1  Potri.010G195000.1  Potri.010G240800.1  Potri.011G040200.1  Potri.011G040300.1  Potri.011G040400.1  Potri.011G041600.1  Potri.011G125900.1  Potri.011G167600.1  Potri.012G039400.1  Potri.012G055600.1  Potri.012G060300.1  Potri.012G072500.1  Potri.012G080400.1  Potri.012G082000.1  Potri.012G084100.1  Potri.012G127700.1  Potri.012G140500.1  Potri.012G140700.2  Potri.013G001000.1  Potri.013G046300.1  Potri.013G056400.1  Potri.013G056500.1  Potri.013G067000.1  Potri.013G067500.1  Potri.013G109300.1  Potri.013G148600.1  Potri.013G149100.1  Potri.013G149200.1  Potri.014G022500.1  Potri.014G035100.1  Potri.014G054700.1  Potri.014G081200.1  Potri.014G100800.1  Potri.014G111200.1  Potri.014G117000.1  Potri.014G122700.2  Potri.015G033600.1  Potri.015G041100.1 | Potri.015G046200.1  Potri.015G067700.1  Potri.015G075600.2  Potri.015G075800.3  Potri.015G077700.1  Potri.015G082700.1  Potri.015G095000.1  Potri.015G129100.1  Potri.015G143400.2  Potri.015G143500.1  Potri.016G099200.1  Potri.016G112300.1  Potri.017G017600.1  Potri.017G071500.1  Potri.017G075000.1  Potri.017G082500.1  Potri.017G085200.1  Potri.017G086300.3  Potri.017G099500.2  Potri.017G112300.1  Potri.017G125600.1  Potri.017G125700.1  Potri.017G125800.1  Potri.017G125900.1  Potri.017G126000.1  Potri.017G128900.1  Potri.017G130300.1  Potri.018G005300.1  Potri.018G049000.1  Potri.018G049200.1  Potri.018G049401.1  Potri.018G049600.1  Potri.018G058800.4  Potri.018G095900.1  Potri.018G127700.1  Potri.019G018200.2  Potri.019G018400.2  Potri.019G036160.1  Potri.019G036340.1  Potri.019G036400.1  Potri.019G040900.1  Potri.019G045900.1  Potri.019G050900.3  Potri.019G081500.1  Potri.019G118200.1  Potri.019G118700.1  Potri.019G118800.1  Potri.019G118900.1  Potri.T011400.1  Potri.T011525.1  ——  ——  —— |

Supplementary Table 3 DEGs in roots, stems and leaves of 84K poplar

| Gene ID | Gene ID | Gene ID | Gene ID |
| --- | --- | --- | --- |
| Potri.001G062500.v4.1 | Potri.002G197900.v4.1 | Potri.005G163700.v4.1 | Potri.010G196400.v4.1 |
| Potri.001G070700.v4.1 | Potri.002G198800.v4.1 | Potri.005G201200.v4.1 | Potri.011G110200.v4.1 |
| Potri.001G158600.v4.1 | Potri.003G054500.v4.1 | Potri.006G071200.v4.1 | Potri.011G110500.v4.1 |
| Potri.001G175400.v4.1 | Potri.003G071000.v4.1 | Potri.006G113400.v4.1 | Potri.013G005700.v4.1 |
| Potri.001G181300.v4.1 | Potri.003G182000.v4.1 | Potri.006G114600.v4.1 | Potri.014G035100.v4.1 |
| Potri.001G288900.v4.1 | Potri.004G019900.v4.1 | Potri.006G122900.v4.1 | Potri.014G146100.v4.1 |
| Potri.001G405500.v4.1 | Potri.004G035100.v4.1 | Potri.006G228400.v4.1 | Potri.016G016300.v4.1 |
| Potri.002G060400.v4.1 | Potri.004G085600.v4.1 | Potri.007G005700.v4.1 | Potri.016G038600.v4.1 |
| Potri.002G060500.v4.1 | Potri.004G089400.v4.1 | Potri.007G142100.v4.1 | Potri.016G105000.v4.1 |
| Potri.002G098100.v4.1 | Potri.004G181700.v4.1 | Potri.008G195700.v4.1 | Potri.018G032200.v4.1 |
| Potri.002G098600.v4.1 | Potri.004G206600.v4.1 | Potri.008G203200.v4.1 | Potri.018G093600.v4.1 |
| Potri.002G114200.v4.1 | Potri.004G207500.v4.1 | Potri.009G005700.v4.1 | Potri.018G094900.v4.1 |
| Potri.002G124600.v4.1 | Potri.004G235400.v4.1 | Potri.009G084000.v4.1 | Potri.018G096028.v4.1 |
| Potri.002G125900.v4.1 | Potri.005G007200.v4.1 | Potri.009G096000.v4.1 | Potri.019G093300.v4.1 |
| Potri.002G128900.v4.1 | Potri.005G087500.v4.1 | Potri.009G168800.v4.1 |  |
| Potri.002G197300.v4.1 | Potri.005G146900.v4.1 | Potri.010G080900.v4.1 |  |

Supplementary Table 4 Cis-element of *PagMYB151*

| Site Name | Sequence | Function |
| --- | --- | --- |
| ABRE | CACGTG | cis-acting element involved in the abscisic acid responsiveness |
| Box 4 | ATTAAT | part of a conserved DNA module involved in light responsiveness |
| CAAT-box | CCAAT | common cis-acting element in promoter and enhancer regions |
| CCAAT-box | CAACGG | MYBHv1 binding site |
| G-Box | CACGTG | cis-acting regulatory element involved in light responsiveness |
| G-box | TACGTG | cis-acting regulatory element involved in light responsiveness |
| GARE-motif | TCTGTTG | gibberellin-responsive element |
| LTR | CCGAAA | cis-acting element involved in low-temperature responsiveness |
| P-box | CCTTTTG | gibberellin-responsive element |
| TATA-box | TACAAAA | core promoter element around -30 of transcription start |
| TCCC-motif | TCTCCCT | part of a light responsive element |
| TCA-element | CCATCTTTTT | cis-acting element involved in salicylic acid responsiveness |

Supplementary Table 5 co-expressession genes of *PagMYB151*

| Gene ID | Gene ID | Gene ID | Gene ID |
| --- | --- | --- | --- |
| Potri.014G096200.v4.1 | Potri.001G187600.v4.1 | Potri.004G185501.v4.1 | Potri.008G007800.v4.1 |
| Potri.014G097900.v4.1 | Potri.001G255100.v4.1 | Potri.004G235400.v4.1 | Potri.008G069400.v4.1 |
| Potri.014G136466.v4.1 | Potri.001G288301.v4.1 | Potri.005G007200.v4.1 | Potri.008G100500.v4.1 |
| Potri.015G018050.v4.1 | Potri.001G288900.v4.1 | Potri.005G107700.v4.1 | Potri.008G192000.v4.1 |
| Potri.015G018200.v4.1 | Potri.001G409500.v4.1 | Potri.005G113600.v4.1 | Potri.009G022500.v4.1 |
| Potri.015G136400.v4.1 | Potri.001G438400.v4.1 | Potri.005G163300.v4.1 | Potri.009G066100.v4.1 |
| Potri.016G117300.v4.1 | Potri.002G004900.v4.1 | Potri.005G163500.v4.1 | Potri.009G073000.v4.1 |
| Potri.017G045150.v4.1 | Potri.002G060400.v4.1 | Potri.005G163600.v4.1 | Potri.009G141400.v4.1 |
| Potri.017G047800.v4.1 | Potri.002G060500.v4.1 | Potri.005G163700.v4.1 | Potri.009G167400.v4.1 |
| Potri.017G077100.v4.1 | Potri.002G098800.v4.1 | Potri.005G176000.v4.1 | Potri.010G078400.v4.1 |
| Potri.017G147200.v4.1 | Potri.002G119300.v4.1 | Potri.005G179900.v4.1 | Potri.010G116000.v4.1 |
| Potri.017G148266.v4.1 | Potri.002G124600.v4.1 | Potri.005G201200.v4.1 | Potri.010G187900.v4.1 |
| Potri.018G061901.v4.1 | Potri.002G128900.v4.1 | Potri.005G201250.v4.1 | Potri.010G191300.v4.1 |
| Potri.018G078801.v4.1 | Potri.002G155300.v4.1 | Potri.005G207600.v4.1 | Potri.011G043300.v4.1 |
| Potri.018G095100.v4.1 | Potri.002G168700.v4.1 | Potri.005G244100.v4.1 | Potri.011G110200.v4.1 |
| Potri.018G095200.v4.1 | Potri.002G181800.v4.1 | Potri.006G062100.v4.1 | Potri.011G129100.v4.1 |
| Potri.018G098200.v4.1 | Potri.002G182100.v4.1 | Potri.006G202200.v4.1 | Potri.011G131100.v4.1 |
| Potri.019G005400.v4.1 | Potri.003G103900.v4.1 | Potri.006G224100.v4.1 | Potri.012G002800.v4.1 |
| Potri.019G014320.v4.1 | Potri.003G150000.v4.1 | Potri.006G226500.v4.1 | Potri.012G007500.v4.1 |
| Potri.019G064700.v4.1 | Potri.003G150100.v4.1 | Potri.006G235500.v4.1 | Potri.012G048700.v4.1 |
| Potri.019G067400.v4.1 | Potri.003G150700.v4.1 | Potri.007G027200.v4.1 | Potri.012G089500.v4.1 |
| Potri.001G025400.v4.1 | Potri.003G150800.v4.1 | Potri.007G038900.v4.1 | Potri.012G134100.v4.1 |
| Potri.001G037000.v4.1 | Potri.004G051800.v4.1 | Potri.007G039000.v4.1 | Potri.013G005700.v4.1 |
| Potri.001G037101.v4.1 | Potri.004G075800.v4.1 | Potri.007G039100.v4.1 | Potri.T125304.v4.1 |
| Potri.001G079800.v4.1 | Potri.004G089400.v4.1 | Potri.007G055500.v4.1 |  |
| Potri.001G158000.v4.1 | Potri.004G181900.v4.1 | Potri.007G142100.v4.1 |  |
