# Supplementary material for: PagMYB151 facilitates proline accumulation to enhance salt tolerance of poplar
Source: BMC Genomics. 2023 Jun 22;24:345. doi: 10.1186/s12864-023-09459-2 (PMC10286439; doi:10.1186/s12864-023-09459-2)
Supplement: Supplementary file 6 — Supplementary Material 6 [file 12864_2023_9459_MOESM6_ESM.docx]

**
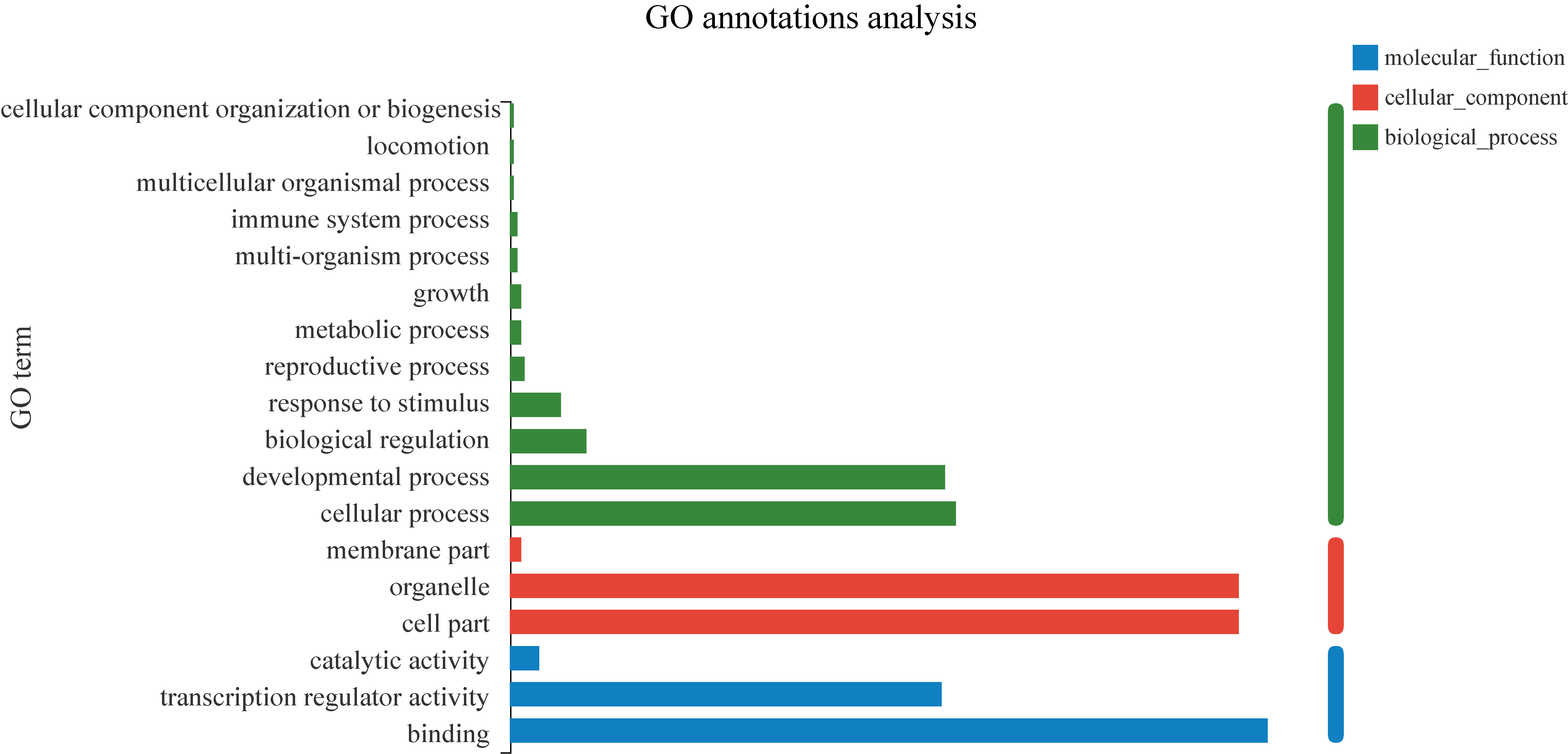
**

**Supplementary Fig.1** GO annotation analysis of R2R3-MYB family.


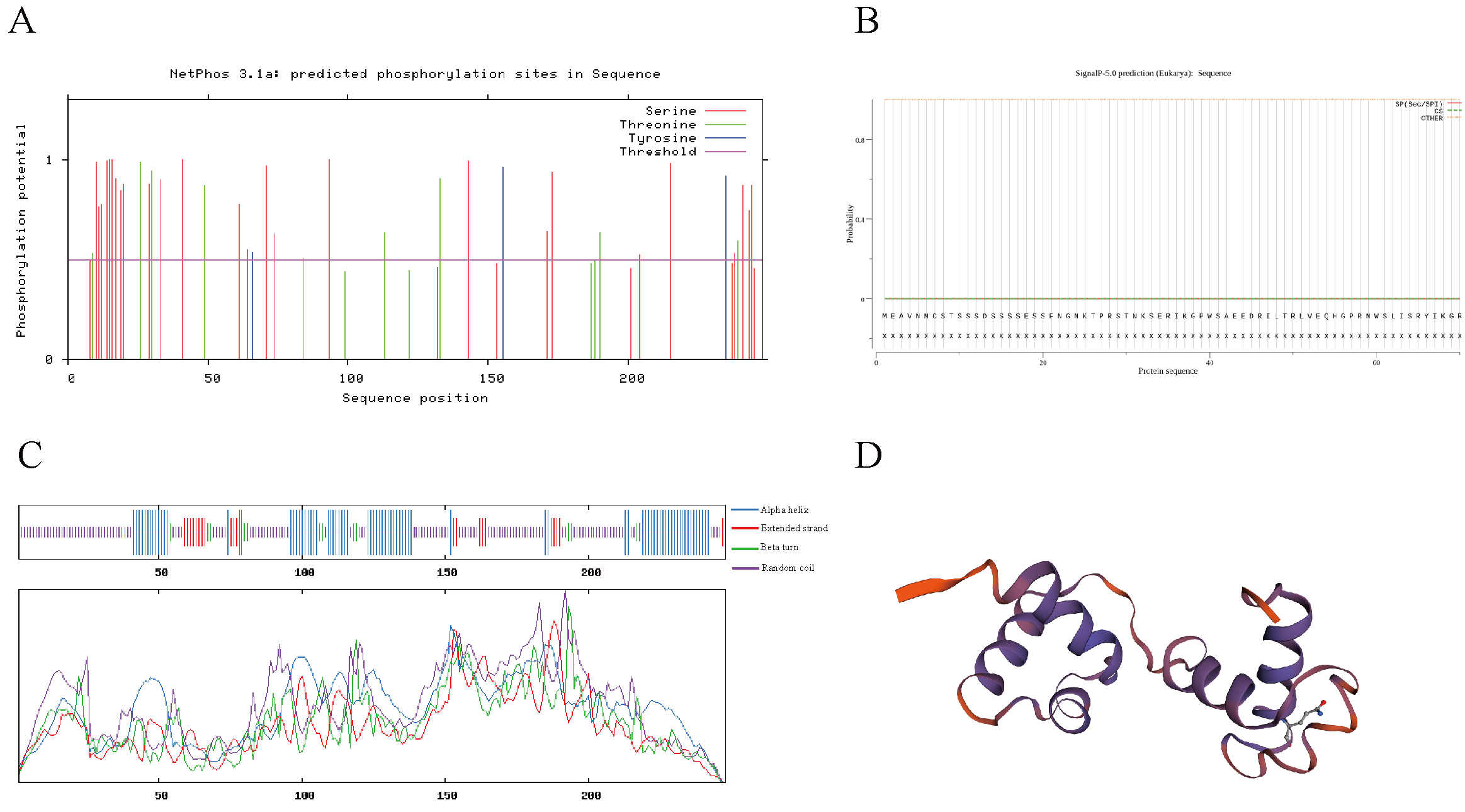


**Supplementary Fig.2** Bioinformatics analysis of PagMYB151 protein. (A) Phosphorylation site prediction (B) Signal peptide prediction (C) Protein secondary structure prediction (D) Protein tertiary structure prediction.


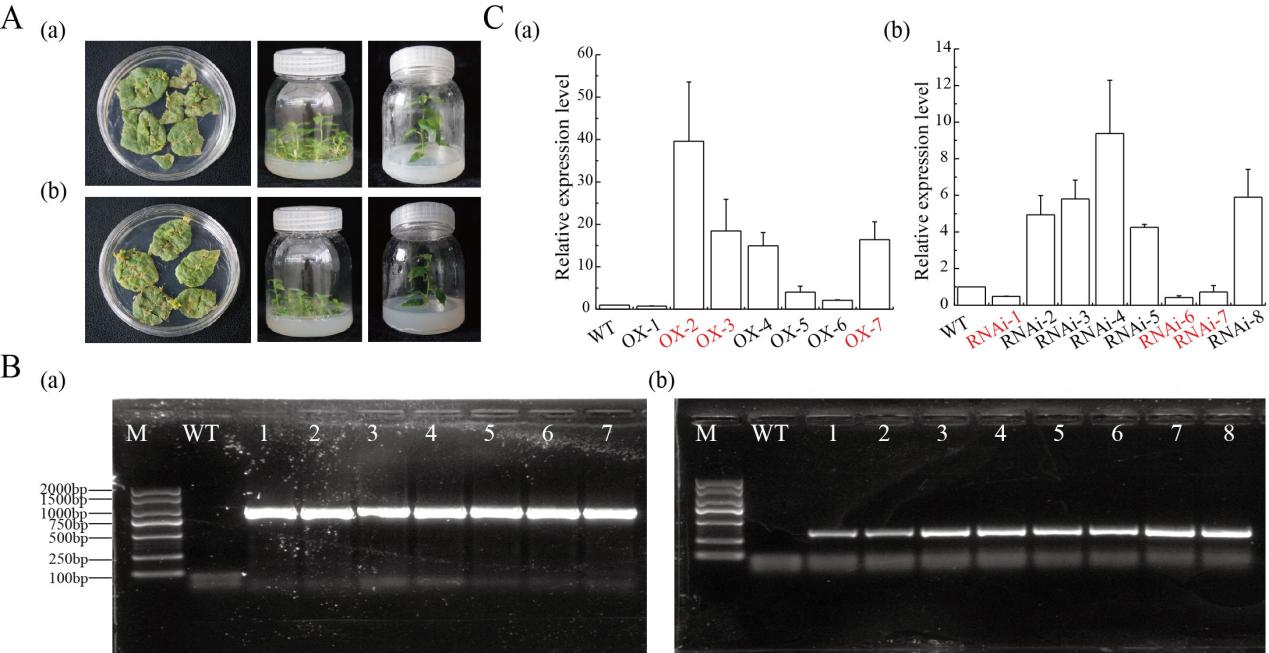


**Supplementary Fig.3** Cultivation and identification of PagMYB151 transgenic plants. (A) Cultivation of PagMYB151 transgenic poplars. Overexpressed lines (a) and (b) suppressed lines. Bars: 1cm. (B) Identification of transgenic lines. (a) Overexpressed lines, (b) Suppressed lines, (C) RT-qPCR identification of transgenic lines. (a ) Overexpressed lines, (b) Suppressed lines.


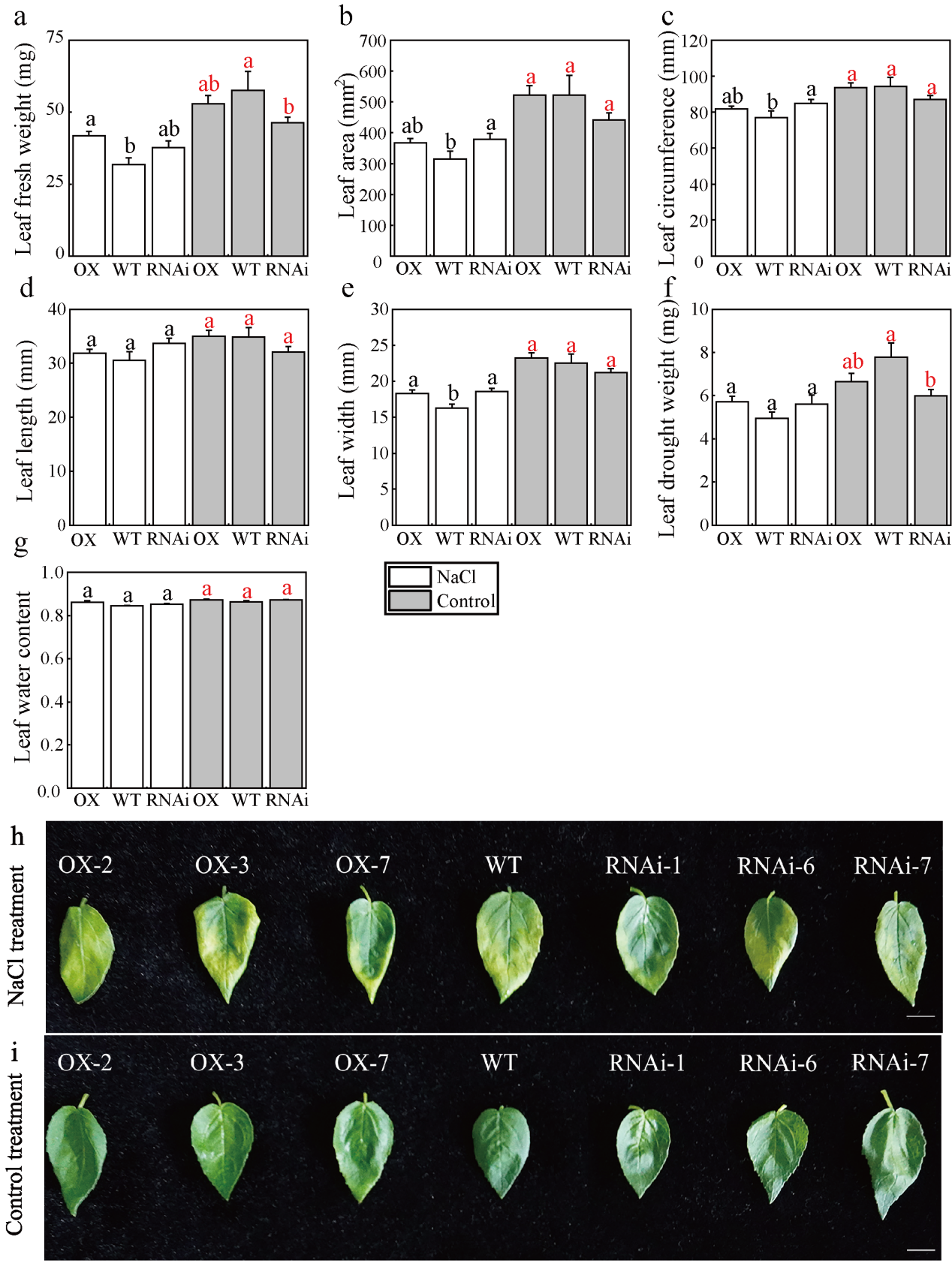


**Supplementary Fig.4** Analysis of leaf index of transgenic and non-transgenic poplars under salt stress. (a) Leaf fresh weight, (b) Leaf area, (c) Leaf circumference, (d) Leaf length, (e) Leaf width, (f) Leaf drought weight, (g) Leaf water content, Leaf morphology under NaCl treatment (h) and control treatment (i) Data represent the means ± SE of 12 independent biological samples of PagMYB151 transgenic poplar (OX-2, OX-3 and OX-7, and RNAi-1, RNAi-6 and RNAi-7 served as three biological replicates, respectively.) and four biological samples of wild-type poplar, respectively. Different letters (red, control; black, NaCl treatment) indicate significant differences at the *P* < 0.05 level.
